# Research Progress of Superconducting Microstrips Single-Photon Detectors

**DOI:** 10.3390/s26216791

**Published:** 2026-10-27

**Authors:** Feiyan Li, Jinji Zheng, Zhiwei Deng

**Affiliations:** 1 School of Transportation Engineering, Nanjing University of Industry Technology, Nanjing 210023, China; lifeiyan13@163.com (F.L.); zhengjinji@niit.edu.cn (J.Z.); 2 School of Electronic Science and Engineering, Nanjing University, Nanjing 210023, China; 3 Jiangsu Autonomous Driving Technology Engineering Center, Nanjing 210023, China

**Keywords:** superconducting materials, single-photon detection, SMSPDs

## Abstract

Superconducting microstrips single-photon detectors (SMSPDs) are a novel type of single-photon sensor based on superconducting thin films patterned into micrometer-scale strip widths. Compared with superconducting nanowire single-photon detectors (SNSPDs), SMSPDs not only substantially lower the fabrication dependence on high-precision electron-beam lithography, but also exhibit lower kinetic inductance, higher bias current, and weaker polarization sensitivity. These advantages pave a new route toward superconducting single-photon detection systems with large active areas and low dark-count rates. Because of their performance, SMSPDs exhibit tremendous application potential in cutting-edge fields such as quantum communication, LiDAR, dark-matter searches, and high-energy particle detection. Accordingly, this paper provides a systematic review of recent advances in SMSPD research, covering the underlying physical mechanisms, material systems, geometrical design, readout circuitry, and application domains. Firstly, this review elaborates on the photon response mechanism of SMSPDs and clarifies its correlation with vortex dynamics. Secondly, this review categorizes the superconducting materials used for SMSPD fabrication and analyzes the influences of characteristic thin-film parameters on device performance. Furthermore, this work concludes the evolution and optimization strategies of geometrical design and readout circuit schemes for SMSPDs, and presents the current application status. Finally, we offer perspectives on its future development trajectory. This review provides a foundation in physical mechanisms and design guidance for the development of large active area SMSPDs with high efficiency and low dark counts.

## 1. Introduction

Single-photon detection is a photodetection technology that senses the energy of individual photons under quantum-limit conditions and converts such energy into measurable electrical signals. As a core sensor operating at the quantum optical limit, single-photon detectors play an irreplaceable role in quantum computing [1,2], quantum key distribution (QKD) [3,4], life sciences [5,6], astrophysics [7,8,9], and LiDAR [10,11,12]. Single-photon detectors are broadly classified into two categories, namely semiconductor single-photon detectors and superconducting single-photon detectors. The former are typically represented by single-photon avalanche diodes (SPADs) and photomultiplier tubes (PMTs) with mature technological routes [13,14]. Nevertheless, restricted by the material bandgap, their core performances such as detection efficiency and dark count rate in the near-infrared to mid-infrared spectral bands have approached fundamental physical limits. In contrast, superconducting single-photon detectors, represented by superconducting nanowire single-photon detectors (SNSPDs) and superconducting microstrip single-photon detectors (SMSPDs) [15,16], leverage their low superconducting energy gaps to achieve fundamental improvements in key metrics such as detection efficiency, dark count rate, response speed, and timing resolution.

In 2001, Gol’tsman et al. experimentally demonstrated for the first time that a NbN superconducting thin film patterned into a nanowire approximately 200 nm wide could achieve single-photon response at 1550 nm, marking the birth of the SNSPDs [17]. SNSPDs are typically fabricated from superconducting thin films with thicknesses of 4–10 nm [18,19,20,21], patterned into meander-shaped nanowire geometries with linewidths of 30–160 nm [22,23,24], operating at temperatures below the superconducting critical temperature, typically in the 2–2.4 K regime [25,26,27]. Over more than two decades of sustained research and engineering advancement, SNSPDs have achieved remarkable comprehensive key metrics: the system detection efficiency (SDE) exceeding 98% [28], timing jitter down to a few picoseconds [29], dark count rate (DCR) below 10^−4^ Hz [30], and maximum count rates reaching the GHz level [31]. However, the superior performance of SNSPDs relies on two critical physical and technological constraints. First, the fabrication of superconducting nanowires with ultra-narrow linewidths relies heavily on high-cost and low-throughput nanofabrication methods such as electron beam lithography (EBL), which features long preparation cycles and hinders large-scale array integration. Second, the active area of a single pixel is merely tens to hundreds of square micrometers, imposing stringent requirements on optical alignment accuracy and limiting its practical applications in multimode fiber coupling, free-space optical paths and large-area detection scenarios.

To address the aforementioned technological bottlenecks, Vodolazov et al. theoretically predicted that superconducting microstrips are capable of single-photon detection under bias current even when the strip width is scaled up to the micrometer scale. The preconditions are that the critical current (*I*_c_) exceeds 70% of the depairing current (*I*_dep_) and the strip width *W* is far smaller than the Pearl penetration depth (*Λ* = 2*λ*^2^/*d*) [32]. Such detectors are termed SMSPDs. Experiments soon revealed definitive single-photon response characteristics in NbN micrometer-width superconducting bridges [33], marking the critical transition of SMSPDs from theoretical prediction to experimental verification. Subsequent studies further verified that SMSPDs can be manufactured using conventional lithography techniques such as laser direct writing and ultraviolet lithography, drastically reducing microfabrication complexity [34]. Benefiting from the linewidth expansion from nanoscale to microscale, SMSPDs exhibit prominent merits in terms of kinetic inductance, current-carrying capacity, polarization insensitivity and effective photosensitive area. Based on these advantages, SMSPDs hold promising application prospects in frontier fields such as quantum information, fundamental physical detection and single-photon imaging, and have gradually evolved into an important research branch in the field of superconducting single-photon detection.

A related review by Zheltikov et al. [35] summarizes the state of SMSPDs, focusing on the vortex detection model, material requirements discussion and performance-enhancement strategies for improving detection efficiency and reducing dark counts. This review presents the effectiveness of various meander topologies in mitigating current crowding and reducing dark-count rates, and compares optical enhancement approaches such as distributed Bragg reflectors (DBR) and resonant cavities. However, it lacks comprehensive content coverage, including summaries of readout circuits and application prospects. Accordingly, this work tracks the state-of-the-art international research progress of SMSPDs and delivers a comprehensive overview covering the mechanisms, materials, structures, readout schemes and applications of SMSPDs. Furthermore, this paper illustrates the developmental history of SMSPDs through comprehensive figures and tables, and discusses the bottlenecks of high-temperature superconductors, the limitations of time-dependent Ginzburg–Landau (TDGL) simulations, and the challenges of multiplexed readout. Therefore, this review complements rather than duplicates the existing one, providing a broader, application-oriented perspective and a practical design reference for high-performance SMSPDs.

This paper is organized as follows. Section 2 elucidates the photon response mechanism of SMSPDs, starting from the hotspot model and emphasizing the dominant role of vortex dynamics in micron-scale-width devices. Section 3 systematically reviews the superconducting material systems for SMSPDs, covering crystalline films, amorphous films, high-temperature superconducting films, and emerging films. Section 4 reviews the underlying mechanisms of detector geometry design and classifies the existing structural configurations. Section 5 focuses on readout circuitry technologies for SMSPDs. Section 6 summarizes the application progress of SMSPDs in frontier fields. Section 7 concludes the review and provides perspectives on the future development directions. This review aims to provide a comprehensive overview of SMSPDs, covering their working mechanisms, material systems, device structures, readout circuits, and application explorations, with the intent of offering potential beginners a fundamental understanding and a quick introduction to SMSPDs.

## 2. Photon Response Mechanism

**Figure 1:**
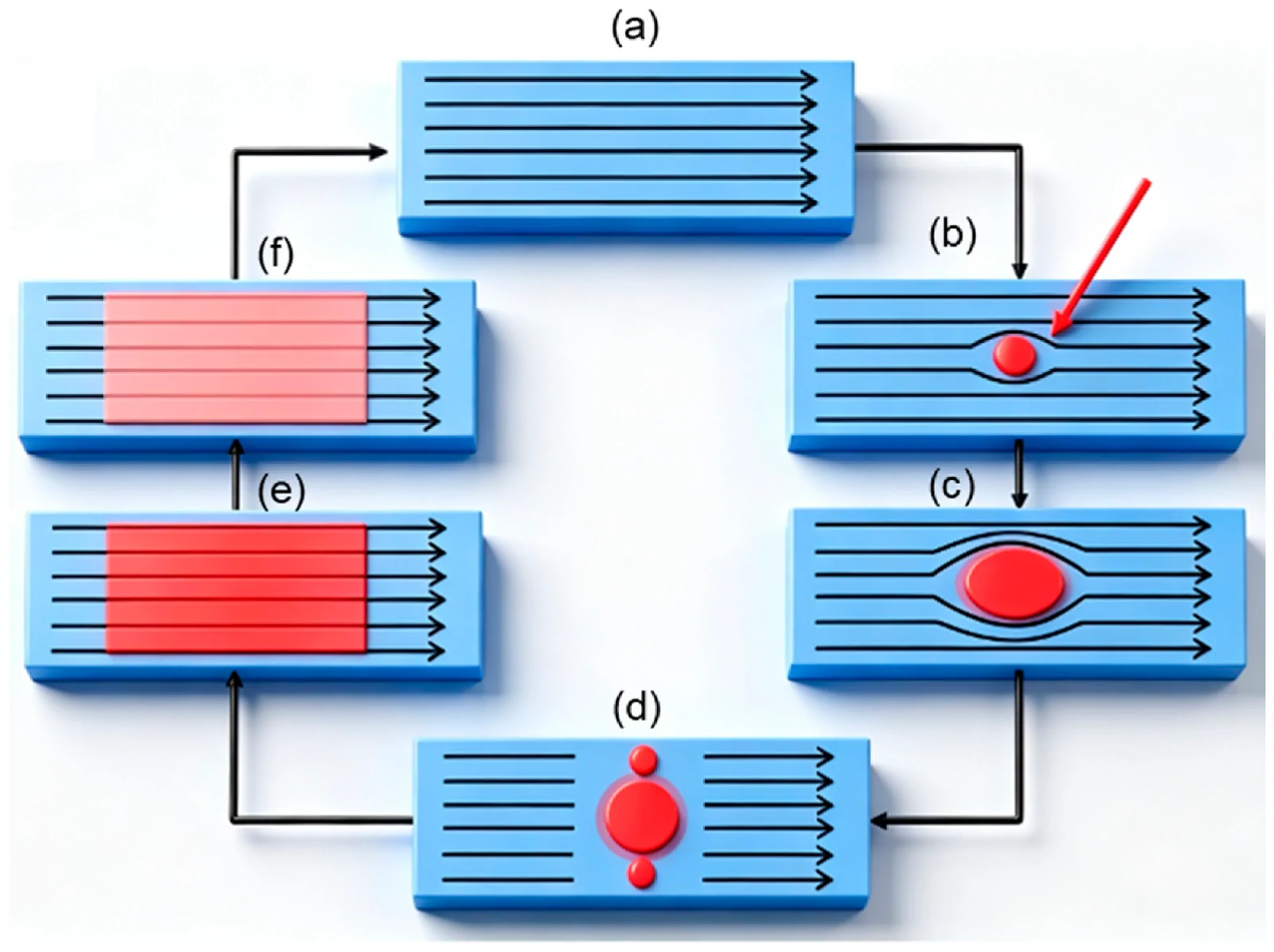
Schematic of the single-photon response process based on the hotspot model: (**a**) The SNSPD is biased near the superconducting critical current. (**b**) The superconducting nanowire absorbs a photon, generating a hotspot. (**c**) The hotspot continues to expand, and the current density on both sides approaches the critical current density. (**d**) The superconducting state near the hotspot is destroyed. (**e**) Under Joule heating, the resistive region propagates, eventually driving the entire nanowire into the normal state. (**f**) The temperature of the nanowire decreases as heat dissipates to the substrate, weakening the Joule heating effect, and the nanowire gradually recovers to the superconducting state.

The hotspot model is commonly employed to phenomenologically explain the photon response mechanism of SNSPDs, and it also plays a supplementary role in understanding the photon response process of SMSPDs. The concept of the hotspot was first proposed by Skocpol et al. in 1974 [36] during their investigation of self-heating effects in superconducting microbridges. The experiment demonstrated that hotspot formation is responsible for the hysteresis observed in the current–voltage (I–V) characteristics of superconducting microbridges. In 2001, Semenov et al. established the hotspot model for the photon response of SNSPDs [37]. This model points out that when a nanowire in the superconducting state absorbs an incident photon, the photon energy far exceeds the superconducting energy gap of the thin film, which is sufficient to break a large number of Cooper pairs and generate a non-uniform distribution of quasiparticles. These quasiparticles, being at a higher temperature than the Cooper pairs, create a localized hotspot within the superconducting film. The formation of the hotspot constricts the effective superconducting cross-sectional area of the nanowire, forcing the superconducting current to be compressed to the residual superconducting regions outside the hotspot. When the superconducting density exceeds the critical current density of the superconducting material, the superconducting state is disrupted, generating a transient voltage response that can be captured by the external readout circuit. The detailed physical evolution of this process is illustrated in Figure 1.

**Figure 2:**
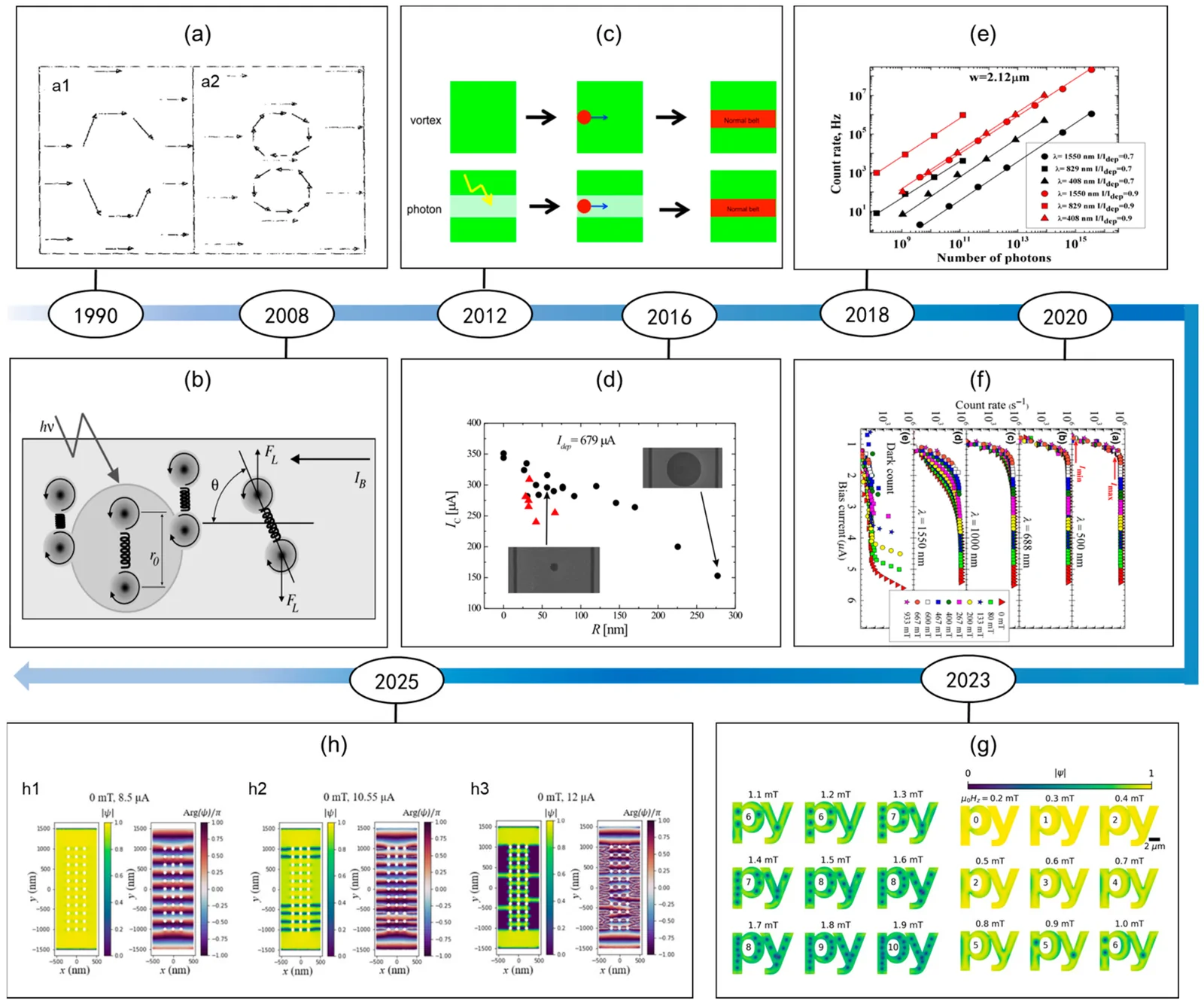
(**a**) Vortex–antivortex pair model [39]. (**b**) Vortex–photon interaction [40]. (**c**) Vortex-assisted photon response model [41]. (**d**) The effect of superconducting microstrip defects on vortex generation [42]. (**e**) Vortex-assisted photon response in NbN superconducting microstrips [33] and (**f**) in MoSi superconducting microstrips [43]. (**g**) The magnitude of the order parameter as a function of uniform applied out-of-plane magnetic field for a superconducting “py” structure [45]. (**h**) The simulation of vortex dynamics in superconducting microstrips with pinholes based on the TDGL equation [46].

The hotspot model typically applies to nanoscale superconducting strips, whereas SMSPDs have micrometer-scale linewidths, and the physical mechanism of their photon response is not yet fully established. However, theoretical and experimental evidence has confirmed that vortex dynamics play a key role in the photon response of SMSPDs. As early as 1990, Kadin et al. [38] discussed vortex dynamics in two-dimensional superconductors, particularly the motion of vortices driven by electrical currents and its effect on resistance. They proposed that a photon-excited vortex–antivortex pair (VAP) could enable photodetection, thereby introducing a novel concept of superconducting devices based on vortex quantum motion. In the same year, Kadin et al. [39] theoretically argued that photon absorption equivalently produces a new VAP to explain the infrared-detection capability of superconducting thin films. Figure 2a shows local photon absorption in a two-dimensional superconducting film, creating a region of suppressed superconductivity on the scale of the vortex-core size *a*_0_. Moreover, the current-diversion distortion induced by photon absorption is electromagnetically equivalent to the generation of a tightly bound vortex-antivortex pair. Under the Lorentz force exerted by the applied bias current, the VAP undergoes depairing to form freely moving vortices, which generate voltage pulses and realize photon detection. Furthermore, Semenov et al. [40] experimentally found that bound VAPs intrinsically exist inside the thin film. Photons serve to lower the energy barrier and trigger the unbinding of the VAPs, rather than generating new ones. Figure 2b sketches the VAP-based single-photon detection scenario for superconducting nanowires. Bound VAPs exist in nanowires under subcritical bias current, and the bias current tends to rotate VAPs into the lowest-energy orientation. Absorption of a photon creates a non-equilibrium spot which locally suppresses the superconducting energy gap and reduces the binding energy of VAPs, facilitating their thermal unbinding. After depairing, the Lorentz force drives free vortices to move apart, generating dissipative normal domains and producing electrical output pulses. In 2012, Bulaevskii et al. [41] compared the two physical processes of thermal dark counts and vortex-assisted photon counts, as shown in Figure 2c. They pointed out that in the vortex-assisted photon count mechanism, incident photons generate local hot spots. These hot-spot regions lower the energy barrier for vortex motion and increase the probability of vortex nucleation and superconducting strip traversal. Driven by the Lorentz force, vortices traverse the superconducting strip. The energy released by vortex motion, together with the energy of the photon-induced hotspot, jointly forms a normal-state belt spanning the entire superconducting strip width.

In 2016, Vodolazov et al. [42] fabricated artificial holes in superconducting strips to investigate the influence of defects on the critical current and vortex generation mechanisms. Figure 2d shows the variation in critical current for superconducting microstrips with holes of different radii etched at their center. When the holes satisfy *ξ* < *R* < *W*, the critical current is independent of the hole radius, where *ξ* is the superconducting coherence length, *R* is the radius of the holes, and *W* is the width of the superconducting microstrip. The experiment revealed that when holes were positioned at the strip center, the formation of vortex–antivortex pairs constituted the dominant mechanism for destroying the superconducting state. In the following year, Vodolazov et al. [32] investigated the single-photon detection mechanism of superconducting microstrips via dynamic equation analysis and verified that superconducting microstrips can achieve single-photon detection under specific operating conditions. The vortex-assisted mechanism for single-photon detection in superconducting microstrips has been experimentally established. In this framework, photon absorption induces local heating (i.e., hotspot formation) within the microstrip, which in turn triggers the generation of vortex–antivortex pairs; this process locally destroys the superconducting state and gives rise to a resistive voltage pulse. This mechanism was first confirmed in NbN microstrips [33]. Figure 2e shows that for a 2.12 μm wide NbN bridge, the photon count rate exhibits a linear relationship with the number of incident photons at different wavelengths and bias currents, with a slope of approximately 1. The experiment demonstrates that micrometer-wide NbN bridges possess single-photon response characteristics. Subsequent studies further extended this investigation to compare the detection response of superconducting strips with different widths [43]. In experiments, 2 µm wide microstrips and 115 nm wide nanowire meanders were fabricated from MoSi films, and the photon count rate was measured as a function of bias current under perpendicular magnetic fields, as shown in Figure 2f. The results revealed a clear distinction between the two geometries: the microstrips exhibited a vortex–antivortex-assisted detection process with strong magnetic-field dependence, whereas the nanowires followed the conventional hotspot model, showing only weak magnetic-field sensitivity.

According to the vortex-assisted single-photon detection, structural modification via artificial holes serves as an effective strategy to optimize the photon response performance of SMSPDs. Comparative experiments on superconducting microstrips with and without hole structures have demonstrated that hole-patterned superconducting microstrips favorably facilitate photon detection, which further confirms the correlation between the photon response characteristics and vortex dynamic behavior of SMSPDs [44]. An open-source Python package, pyTDGL, was introduced to solve the generalized TDGL equations through a finite-volume method. It enables simulations of vortex and phase dynamics in two-dimensional superconducting thin films of arbitrary geometry, and its applications were demonstrated for mesoscopic superconducting devices [45]. As an example, Figure 2g presents simulation results for the order-parameter magnitude of a superconducting “py”-shaped film under different out-of-plane applied magnetic fields. TDGL simulations provide space- and time-resolved information on the superconducting order parameter *ψ*(r,t), including its amplitude and phase, as well as the supercurrent and normal current densities, the electric potential, and the magnetic field distribution in thin-film superconductors of arbitrary geometry. They can directly visualize vortex nucleation, vortex–antivortex pair generation, vortex flow, pinning, and phase slips, and can be used to calculate current–voltage characteristics and critical currents as functions of applied magnetic field. However, TDGL is a phenomenological model with several limitations for real-device prediction. The model is applicable near the critical temperature and for dirty superconductors where the inelastic diffusion length is much smaller than the coherence length. It generally neglects Joule heating and thermal latching, and adopts idealized boundary conditions together with uniform material parameters. Thus, TDGL simulation results can serve as qualitative references for device design, and the actual performance of devices must be validated by experiments.

Based on the pyTDGL, the vortex dynamics and their effect on the critical current have been systematically investigated in superconducting microstrips patterned with periodic rectangular pinhole arrays, and WSi-based microstrips with three different pinhole spacings consistent with the simulation configurations were fabricated [46]. Figure 2h displays the simulated amplitude and phase of the superconducting order parameter for a pinhole-patterned superconducting microstrip under zero external magnetic field at three bias currents. The simulation experiment visually illustrates the three-state evolution of the superconducting microstrip, from the vortex-free Meissner state and vortex-flow mixed state to the resistive state. Furthermore, by tuning the longitudinal spacing of the superconducting microstrip with periodic pinhole arrays, the experiment measured the critical current of the device under different perpendicular external magnetic fields and obtained the critical current (*I*_c_)–magnetic field (*B*) curve data. The results revealed a distinct double-plateau feature in the *I*_c_(*B*) curves of the microstrips with periodic pinhole arrays, as well as the observed trend of increasing critical current with decreasing pinhole spacing. This study provides a viable strategy for optimizing dark-count suppression and critical-current performance in SMSPDs. In addition to structural engineering, surface oxidation can also affect the vortex flow instability and vortex dynamics of superconducting microstrips [47]. Thus, the oxidized superconducting thin films are incompatible for the fabrication of SMSPDs due to degraded device photon response.

Numerous studies have demonstrated that vortices play an essential role in the photon response mechanism of SMSPDs. Under the combined influence of bias current and incident photons, vortex motion within the superconducting microstrip dominates both energy dissipation and the destruction of the superconducting state. Vortex motion can be categorized into two scenarios: single-vortex traversal and vortex–antivortex depairing [41]. Single-vortex traversal refers to the process in which the energy of an incident photon drives a single vortex to move from one edge of the superconducting film to the opposite edge, thereby destroying superconductivity along its path and generating energy dissipation. In contrast, vortex–antivortex depairing occurs when photon absorption suppresses the superconducting order parameter and reduces the energy barrier for vortex crossing. Under the applied bias current, the vortex–antivortex pairs dissociate and move in opposite directions, causing the thin film to transition from the superconducting to the normal state.

Notably, the photon response relies primarily on fluctuation effects to induce the transition from the superconducting to the normal state. During the phase transition, an ultrafast transient voltage pulse can be detected across the superconducting strip. There are two types of thermally excited fluctuations. One is the fluctuation of quasiparticle density, which macroscopically manifests as local fluctuations in the critical current density. When the local current density exceeds the critical value, the device transiently enters the normal state. The other is the fluctuation of confined vortices. Under the action of the Lorentz force, the confined vortex–antivortex pairs can dissociate at relatively high current densities and move toward the edges of the strip [46]. Generally, the interaction between incident photons and the superconducting strip is a complex process. Thus, two fluctuation events are not independent; that is, both quasiparticle diffusion and vortex motion play facilitating roles in the photon detection event [48].

## 3. Superconducting Materials for SMSPDs

The detection performance of SMSPDs is governed by the physical parameters of the superconducting thin-film materials, including the superconducting critical temperature (*T_c_*), superconducting energy gap (*Δ*), kinetic inductance (*K*), normal-state sheet resistance (*R_sheet_*) and electron diffusion coefficient (*D*). Typically, the superconducting critical temperature and energy gap are correlated with the device detection efficiency (SDE) and quantum efficiency (QE) of detectors, such that lower values of these two parameters contribute to enhanced SDE and QE. Furthermore, low diffusion coefficient and kinetic inductance can effectively shorten the recovery time, thereby improving the detector counting rate. In addition, superior thin-film uniformity enhances the consistency of large-area devices, enabling the bias current to approach the critical current more closely while suppressing dark counts. Lastly, excellent process compatibility of the thin film facilitates micro/nano-fabrication integration, which is essential for the practical implementation of SMSPD devices.

As SMSPD research progresses from fundamental performance characterization toward application-oriented system integration, extensive efforts have been devoted to exploring and optimizing a variety of superconducting thin-film materials. To date, the superconducting thin-film materials employed in the SMSPD field can be broadly categorized into crystalline thin films, amorphous thin films, high-temperature superconducting films, and other emerging superconducting materials. Collectively, these material systems are driving the continuous evolution of SMSPD technology toward higher performance and broader application domains. Table 1 summarizes the main superconducting materials used for SMSPD fabrication and their key parameters.

### 3.1. Crystalline Materials

As the earliest crystalline superconducting material used for fabricating SMSPDs, NbN thin films have laid an important foundation for the development of detector technology, owing to their well-established fabrication processes and superconducting properties. Experimental studies have verified that high sheet resistance serves as a key factor in elevating the saturation plateau of detection efficiency for SMSPDs [49]. The underlying physical mechanism is that a higher sheet resistance corresponds to a lower quasiparticle diffusion coefficient, which facilitates the maintenance of stable vortex motion within the localized region, thereby enhancing the photon–vortex coupling efficiency. Here, the sheet resistance of NbN films can be tuned by adjusting film thickness and deposition parameters. Similarly, SMSPDs fabricated from 5 nm thick high-resistivity NbN films (with sheet resistance ≈ 1 kΩ/□ and Tc ≥ 8 K) have been experimentally confirmed to exhibit saturated detection efficiency in both the telecommunications band and a broader spectral range [51].

**Figure 3:**
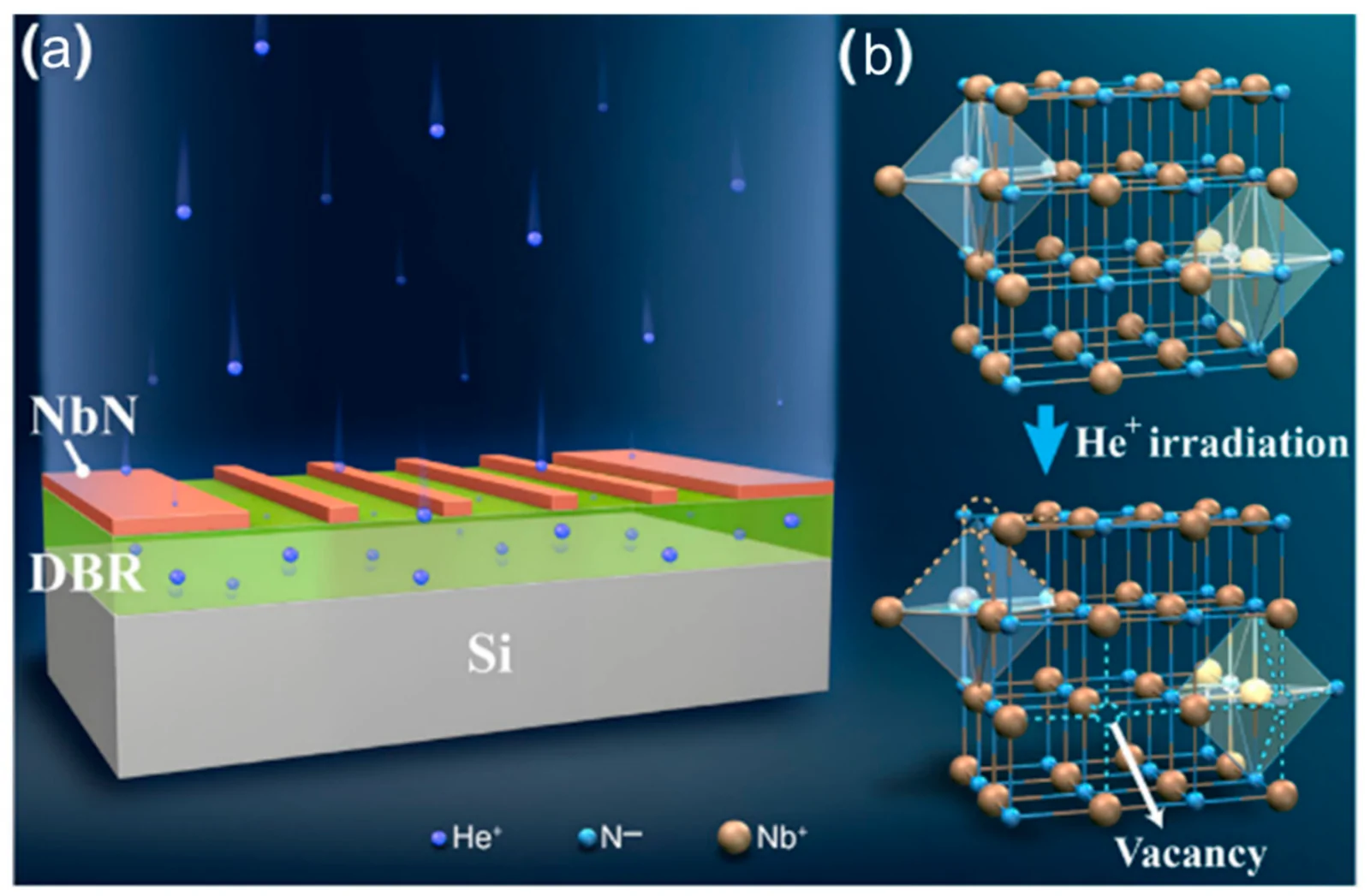
(**a**) Schematic of the He ion irradiation process on NbN film. (**b**) Comparison of NbN crystal structures before and after He ion irradiation [62].

Additionally, ion irradiation provides an effective technique to modulate the properties of superconducting films. The irradiation process introduces defects such as oxygen vacancies and lattice disorder into the thin films, which in turn can change the physical properties by reducing the density of electronic states at the Fermi level (*N*_0_), as well as altering the superconducting energy gap and the electron diffusion coefficient. The schematic of the He-ion irradiation process on NbN film is shown in Figure 3. Ion irradiation has been applied to NbN thin films, enabling effective modulation of the superconducting critical temperature and quasiparticle diffusion coefficient [50]. After He-ion irradiation, the critical temperature of 7 nm thick NbN films decreased from 7.1 K to 6.4 K, the sheet resistance increased from 839 Ω/□ to 1036 Ω/□, and the ratio of switching current to depairing current improved from 0.63 to 0.66. The ion-irradiated NbN SMSPDs achieved a system detection efficiency (SDE) of 92.2% at the 1550 nm wavelength. Ion irradiation is an effective post-processing tuning approach that can significantly improve the system detection efficiency of the device. However, it is an additional post-processing step added to the original fabrication flow, which increases process complexity and imposes stringent requirements on the ion beam dose, irradiation uniformity across the film, and equipment conditions. These demanding constraints often affect the device yield.

In recent years, NbTiN superconducting films have also been employed for SMSPD fabrication. NbTiN films combine a relatively high critical temperature, good film uniformity, and compatibility with photolithographic processes, making them a promising candidate for large-area devices. SMSPDs with a strip width of 1 μm and an active area of 420 × 420 μm^2^ have been fabricated via ultraviolet photolithography [63]. The detector demonstrated single-photon sensitivity at 1550 nm wavelength, and the detection efficiency exhibited a saturation trend with increasing bias current in the 406–850 nm spectral range. Furthermore, systematic tests on NbTiN-based single-photon detectors with strip widths ranging from 0.1 μm to 30 μm prove that single-photon response and efficiency saturation can be realized across all sizes [57]. Nevertheless, the deviation between switching current and depairing current rises gradually as the strip width increases, which is mainly attributed to uneven bias current distribution concentrated at strip edges.

By controlling the ion dose and irradiation region, a gradient distribution of the critical current across the superconducting strip can be achieved. This approach can avoid the current crowding effect that typically occurs at the edges of conventional uniformly patterned strips. To enable wide superconducting strips to achieve efficient single-photon detection while simultaneously suppressing intrinsic dark counts, a superconducting strip bank structure with high critical current (HCCB) has been proposed [64]. Here, Ar-ion irradiation is applied to the central region of NbTiN superconducting strips to reduce their critical current per unit width. Consequently, the edge regions have a higher critical current per unit width than the central region. This design suppresses vortex penetration and intrinsic dark counts, enables uniform high bias current distribution in the central zone, and resolves the edge failure problem existing in wide superconducting strips. When a 20 µm wide HCCB structure was integrated with an optical cavity, the detector achieved a system detection efficiency of approximately 78% at the 1550 nm wavelength.

### 3.2. Amorphous Materials

**Figure 4:**
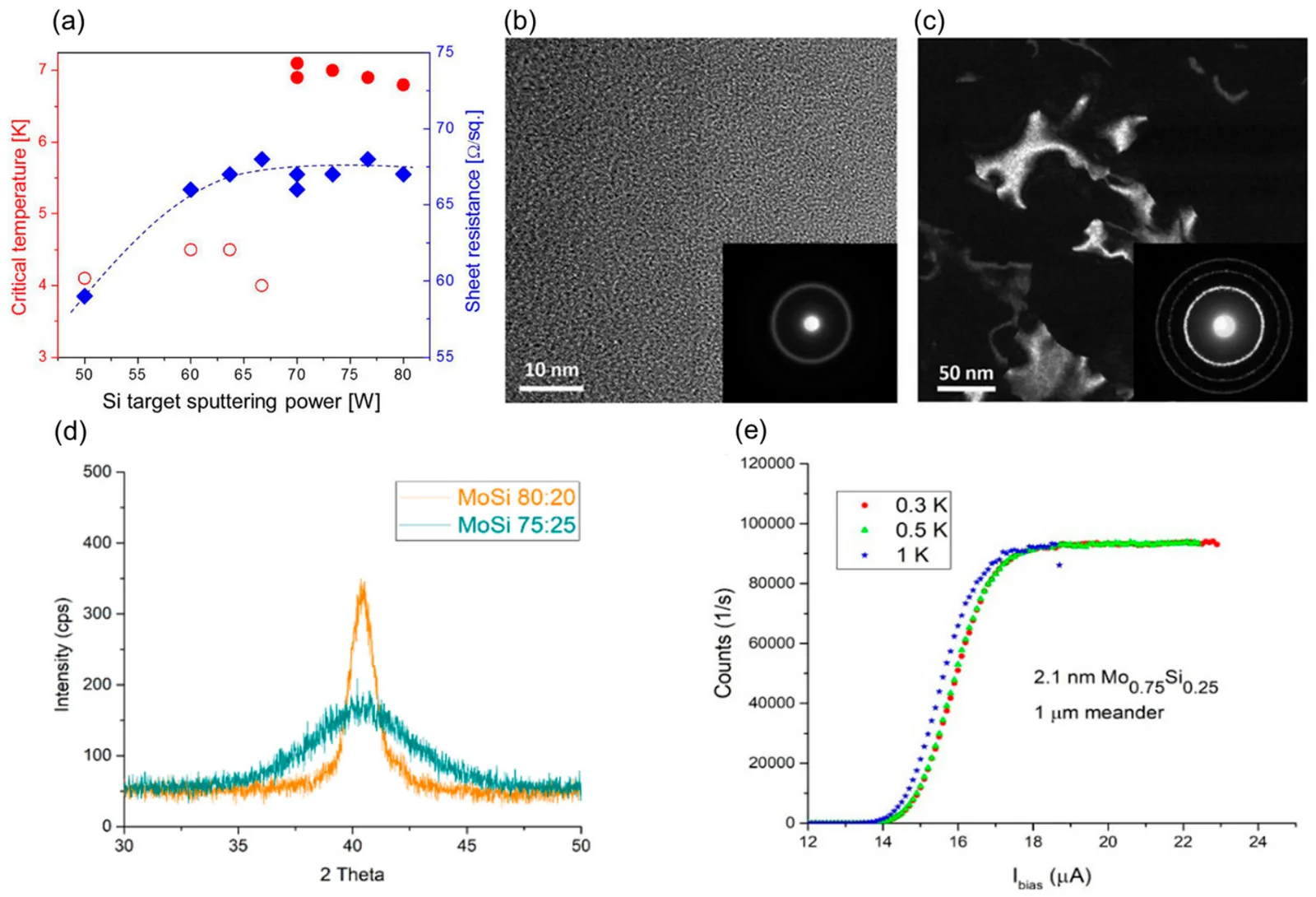
(**a**) Variation in critical temperature and sheet resistance versus silicon content in 20 nm MoSi thin films. (**b**) A dark-field image and diffraction pattern of the MoSi film sputtered at 73 W to the Si target and (**c**) with 50 W applied to the Si target [55]. (**d**) Grazing incidence X-ray diffraction measurements for the two different composition MoSi films; the sharper peak at 2θ = 40° indicates higher crystalline content in the Mo_0.80_Si_0.20_ film. (**e**) The saturated count rates for SMSPD made from the Mo_0.75_Si_0.25_ thin film at different temperatures [56].

Amorphous thin films occupy an important position in the material system of SMSPDs due to their intrinsic physical properties. Compared with crystalline thin films, amorphous superconducting thin films possess intrinsic advantages such as a low superconducting energy gap, excellent structural uniformity and a low quasiparticle diffusion coefficient. These properties facilitate the photoresponse capability and reduce sensitivity to local structural defects, providing a material foundation for large-scale fabrication of detectors. Among them, MoSi and WSi thin films are the two most representative amorphous materials [52,55]. SMSPDs based on amorphous superconducting thin films exhibit saturated photon counting, with quantum efficiency approaching unity [65]. Interestingly, the growth conditions of thin films have a direct impact on their superconducting critical temperature, sheet resistance and amorphous morphology. As illustrated in Figure 4a, the critical temperature of MoSi films varies markedly with Si-target sputtering power, whereas their sheet resistance remains nearly constant within a specific range of Si-target sputtering power. As shown in Figure 4a,b, variations in Si target sputtering power induce structural changes in MoSi thin films. Compared with the sputtering power of 73 W, the sharp ring with distinct spots in the diffraction pattern obtained at 50 W indicates a polycrystalline structure with large-sized grains.

As revealed by Figure 4a–c, it can be seen that the morphological characteristics of thin films are correlated with their performance parameters. Specifically, amorphous or poorly crystallized films exhibit higher critical temperatures, whereas films with large-sized grains possess relatively lower critical temperatures. Furthermore, the critical temperature in turn determines key performance metrics of devices. Currently, amorphous superconducting thin films have become a mainstream research direction in the field of SMSPDs. Researchers optimize film performance through composition modulation, aiming to develop amorphous films that can simultaneously satisfy the requirements for high-efficiency single-photon detection, large-area fabrication, and good integration compatibility.

Lita et al. [56] found that the stoichiometric ratio of Mo and Si in MoSi films affects the detection efficiency of the devices, as illustrated in Figure 4c,d. A lower Si content in the film tends to cause film crystallization problems, making it difficult to achieve saturated detection efficiency. For example, Mo_0.8_Si_0.2_ films are susceptible to spontaneous phase transformation and precipitation of Mo_3_Si grains with increasing thickness, which broadens the superconducting transition and inhibits the saturation of detection efficiency. In contrast, Mo_0.75_Si_0.25_ films with a higher Si content retain a stable amorphous structure. Therefore, by tuning the Si content and film thickness of MoSi films, the single-photon detection performance of the devices can be optimized. Similarly, Luskin et al. [53] compared SMSPDs fabricated from WSi films with three different stoichiometric ratios, and found that adjusting the W:Si ratio can change the physical parameters of the films. In the films, higher silicon content leads to lower critical temperature and higher sheet resistance. Experimental results prove that W_42_Si_58_ films are the optimal choice for the fabrication of high-performance arrayed SMSPDs.

### 3.3. High-Temperature Superconducting Materials

**Figure 5:**
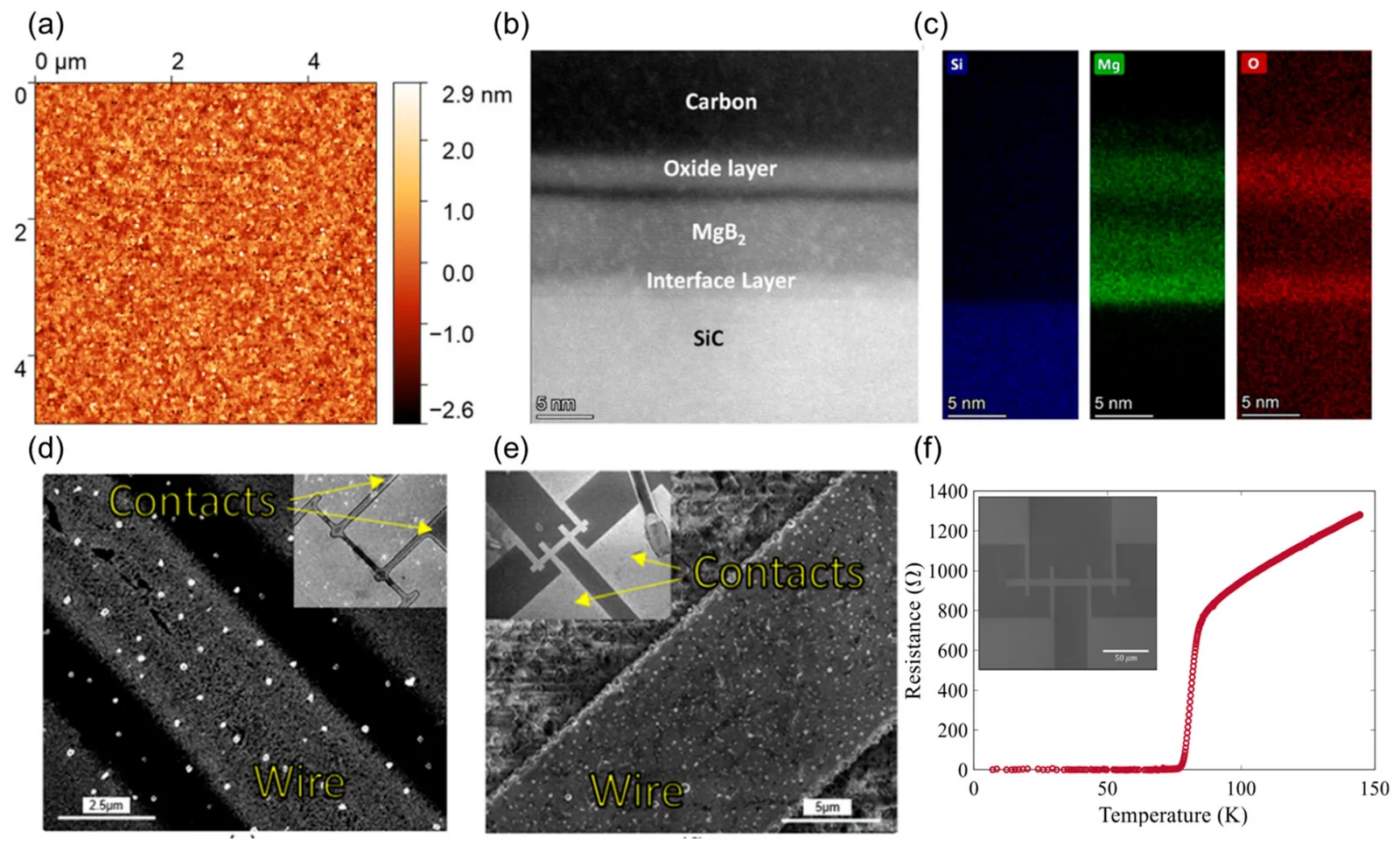
(**a**) Atomic-force microscope image of the surface topography of MgB_2_ thin film. (**b**) Cross-section of MgB_2_ thin film by scanning transmission electron microscopy. (**c**) Elemental characterization of Si, Mg, and O using energy-dispersive X-ray spectroscopy [60]. (**d**) YBCO microstrip by pulsed laser deposition and wet etching method. (**e**) YBCO microstrip by SEG process [61]. (**f**) The temperature-dependent resistance for a 50 nm thickness YBCO microstrip fabricated by the SEG method [67].

Although amorphous superconducting thin films are widely used in SMSPDs, their low superconducting critical temperature leads to a strong reliance on cryogenic cooling systems (<2 K), which limits their practical applications. High-temperature superconducting thin films for SMSPD fabrication have gradually attracted attention in recent years, enabling detector operation above 4.2 K. Among high-temperature superconducting films, MgB_2_ thin films exhibit a relatively high superconducting critical temperature and low kinetic inductance, making them a candidate material for high-temperature, fast-response devices [66]. However, large-area growth of MgB_2_ films often suffers from inhomogeneity, which remains a key technical challenge. To address this issue, Charaev et al. [60] employed hybrid physical–chemical vapor deposition (HPCVD) to grow high-quality MgB_2_ superconducting thin films, patterned them into superconducting microstrips, and irradiated the microstrip surfaces with helium ions. The material characterizations of the MgB_2_ thin film are shown in Figure 5a–c. Experimental results showed that the MgB_2_-based superconducting microstrips can operate at temperatures up to 20 K, with a photosensitive area of 400 × 400 µm^2^ and a recovery time as short as 1 ns. In addition, the high-temperature superconducting film YBCO has emerged as a promising candidate for superconducting single-photon detectors. As shown in Figure 5d,e, by comparing the fabrication methods of YBCO microstrips, high-quality YBCO superconducting microstrips have been successfully fabricated via a selective epitaxial growth (SEG) process, which avoids the degradation of superconducting properties caused by conventional fabrication techniques [61]. Subsequently, a superconducting YBCO microstrip was demonstrated to achieve stable ultrafast photon response at 76 K, with a timing jitter of approximately 100 ps. As shown in Figure 5f, the critical temperature of the YBCO microstrip is 81.5 K, which provides a basis for developing YBCO-SMSPD devices with higher operating temperatures [67]. Furthermore, the photon response physical mechanism of superconducting YBCO microstrips was analyzed, and a statistical model for hotspot generation was established and validated. Using the same fabrication method, the photon response wavelength of YBCO superconducting microstrips has been extended to the mid-infrared region, achieving detection in the 3.25–4.25 µm range at an operating temperature of 80.5 K [68].

Despite the promising potential for high-temperature operation of high-temperature superconductors such as MgB_2_ and YBCO, several critical technical bottlenecks remain. Film inhomogeneity originating from fabrication processes, elemental diffusion and ion modification give rise to local weak links, which limit the yield and uniformity of large-area devices. Detectors based on these high-temperature materials exhibit low detection efficiency. Although helium-ion-irradiated MgB_2_ microstrips achieve saturated intrinsic detection efficiency at low temperatures, their system detection efficiency remains unsatisfactory. By contrast, the photoresponse of YBCO microstrips is predominantly governed by bolometric thermal effects instead of nonequilibrium hot-spot-triggered single-photon transitions, which restricts the intrinsic conversion efficiency. Moreover, these devices generally suffer from large time jitter. YBCO microstrips can reach a jitter of up to 100 ps under optimal conditions, and jitter further degrades under low-photon-flux conditions. Owing to the above-mentioned challenges, the detectors based on high-temperature superconductors cannot yet match the overall performance of conventional low-*T*_c_ SNSPDs.

Furthermore, researchers have developed fabrication techniques for microstrips based on the iron-based high-temperature superconductor NdFeAs(O,H). By optimizing etching conditions, the smoothness of the NdFeAs(O,H) microstrips was improved, offering an alternative material choice for high-temperature superconducting single-photon detectors [69]. These studies provide an important material foundation for advancing SMSPDs toward practical applications. However, the detection efficiency and timing jitter performance of SMSPDs based on high-temperature superconducting films still require further improvement.

### 3.4. Emerging Superconducting Materials

In the continuous exploration of superconducting thin-film material systems for SMSPDs, emerging materials such as NbRe and NbReN have gradually attracted research attention in recent years. Distinct from amorphous films, which typically demand millikelvin operating temperatures, ultrathin NbRe films exhibit polycrystalline nanograined morphology with grain sizes of several nanometers, delivering an intermediate superconducting energy gap between conventional crystalline Nb-based superconductors and fully amorphous superconducting alloys. Such material features endow NbRe with a relatively high critical temperature and a low quasiparticle relaxation rate, providing new pathways toward elevated operating temperature, fast response speed, and extended spectral detection range for SMSPD devices.

In 2022, Cirillo et al. [70] confirmed that ultrathin NbRe microstrips exhibit hysteretic I–V characteristics, which satisfy the basic material requirements for superconducting single-photon detectors. In the same year, superconducting microstrips were fabricated from disordered NbRe films [58]. Experiments verified that 4 nm thick NbRe microstrips can realize single-photon detection at 1550 nm under 1.79 K, with a threshold bias current comparable to that of amorphous superconducting materials. Furthermore, Cirillo et al. [71] extended the response wavelength of NbRe-based SMSPDs, and the devices achieved single-photon sensitivity at both 1.55 μm and 2 μm under an operating temperature of 1.6 K. Similar to NbRe, NbReN films also exhibit a low quasiparticle relaxation rate and a low superconducting energy gap. The quasiparticle relaxation time of the films has been found to correlate with pinning properties and geometric dimensions. Researchers have fabricated NbReN films into superconducting microstrips and confirmed their great potential for superconducting single-photon detectors [59]. At the same time, NbReN also faces obvious challenges for SMSPD development. Strong bulk pinning inside the films reduces the vortex critical velocity, while edge defects introduced by photolithography lower the vortex-entry barrier, both of which deteriorate device performance metrics. Further optimization of deposition conditions and microfabrication processes is required to improve edge quality of microstrips and suppress undesirable pinning centers, so as to exploit the material advantages of NbReN for high-performance SMSPDs.

Compared with amorphous MoSi/WSi SMSPDs that mostly operate below a temperature of 1 K, both NbRe- and NbReN-based devices can work above 1.6 K, which greatly relaxes the requirements for cryogenic systems. However, their performance is extremely sensitive to film stoichiometry, microstructure and patterning quality. These material-process-related uncertainties remain major obstacles to large-scale applications of NbRe-family SMSPDs.

## 4. Geometry Structures for SMSPDs

**Figure 6:**
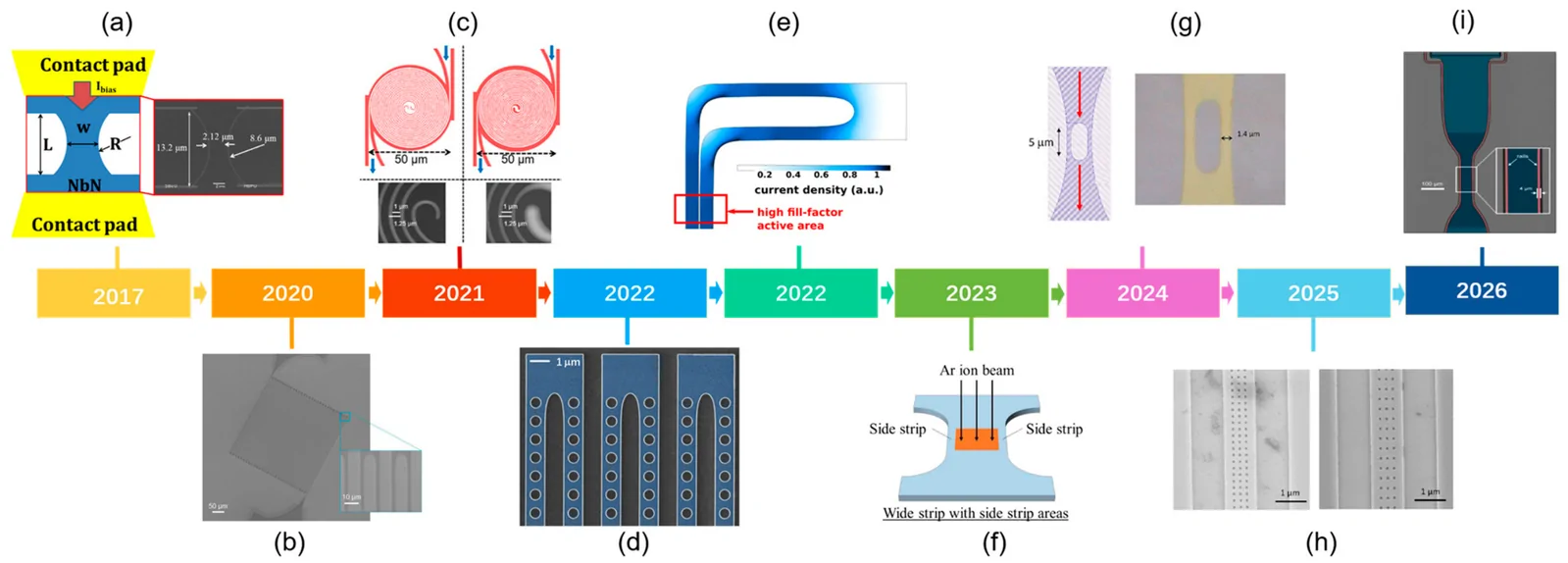
The evolution of geometric structures for SMSPDs. (**a**) Superconducting microbridge [33]. (**b**) Meandering superconducting microstrip [55]. (**c**) Spiral superconducting microstrip [50]. (**d**) Hole-patterned superconducting microstrip [44]. (**e**) Superconducting microstrip with a candelabra-style bend [72]. (**f**) Superconducting wide strip with HCCB [64]. (**g**) A pair of parallel superconducting microstrip [71]. (**h**) Superconducting microstrip with pinholes [46]. (**i**) Superconducting microstrip (blue) integrated with adjacent rails (pink) [73].

The meandering superconducting microstrip is a typical structure of SMSPDs, in which the superconducting microstrip is arranged in a serpentine shape with a certain linewidth and filling factor. On the basis of the meander geometry, systematic architectural optimizations of SMSPDs have been conducted, including geometrical configuration design, local material-property modulation, and topological structure innovations, so as to fully exploit the device’s advantages in large active area, temporal response, and polarization insensitivity. The evolution of these structural designs is illustrated in Figure 6. In this section, this review summarizes the types of geometrical structures of SMSPDs and discusses the correlation between device geometry and the underlying physical mechanisms.

### 4.1. Current Crowding Effect and Geometric Structure Optimization

Superconducting microbridge structures are typically adopted for experimental verification of their single-photon response capability, as illustrated in Figure 6a. Afterward, SMSPDs employ meandering superconducting microstrip geometries to expand the active area of devices, as shown in Figure 6b. In conventional meander structures, the superconducting current tends to accumulate locally at the bends due to abrupt changes in the current path curvature. The current crowding triggers an early transition from the superconducting to the normal state, resulting in a superconducting critical current far below the theoretical depairing current and thus limiting the improvement of detection efficiency. To relieve the current crowding effect, researchers optimized the design by rounding the corners at the meander bends or by increasing the film thickness in the bend regions [74,75]. Since the bent parts of meandering layouts are located inside the active detection region, they restrict the upper limits of filling factor and critical current of superconducting microstrips. Moreover, a low filling factor produces an obvious grating effect, which makes the device polarization-sensitive to incident photons.

To overcome the current crowding at bent regions, Xu et al. [50] designed spiral-shaped superconducting microstrips, as shown in Figure 6c. The gradual curvature enables uniform current distribution at bends and avoids local current crowding. Experimental results show that the switching current of the double-spiral structure reaches 65.2 μA, which is 1.5 times that of the traditional meandering structure and close to the theoretical depairing current. Furthermore, to optimize the polarization sensitivity and fill factor of the conventional meander structure, a “candelabra-style” meander structure has been proposed [75], as shown in Figure 6e. In this design, all bend sections are relocated outside the active photosensitive region, leaving only parallel straight microstrips within the active area. This configuration effectively eliminates the adverse impact of current crowding on detection efficiency, while achieving a filling factor of 0.91, a system detection efficiency of >91.8% at 1550 nm, and a polarization sensitivity as low as 1.02 ± 0.008. This candelabra architecture has been further employed to fabricate large-area SMSPDs with a filling factor of 0.8, which were successfully integrated into a free-space-coupled time-of-flight imaging system [76]. Kong et al. [77] further integrated the candelabra structure with a high-kinetic-inductance design of superconducting microstrips, enabling the device to achieve up to 10-photon resolution and broadening the application potential of SMSPDs in the field of quantum information.

The current inhomogeneity in superconducting microstrips not only degrades detection efficiency but also elevates the dark count rate. Especially when the strip width exceeds 20 μm, bias current tends to concentrate at the edges due to the edge effect, significantly increasing the intrinsic dark counts. To address this issue, Yabuno et al. [64] proposed an HCCB structure, in which the central region of the superconducting microstrip is selectively irradiated with argon ions to reduce its local critical current density, as shown in Figure 6f. Consequently, the critical current per unit width at the edges is higher than that in the central region, which suppresses vortex penetration from the edges and thus reduces dark counts. The wide-strip SMSPD based on the HCCB structure achieved a polarization-insensitive system detection efficiency of approximately 78% at 1550 nm, with a system dark count rate of 80 Hz and a timing jitter of 29.8 ps.

**Figure 7:**
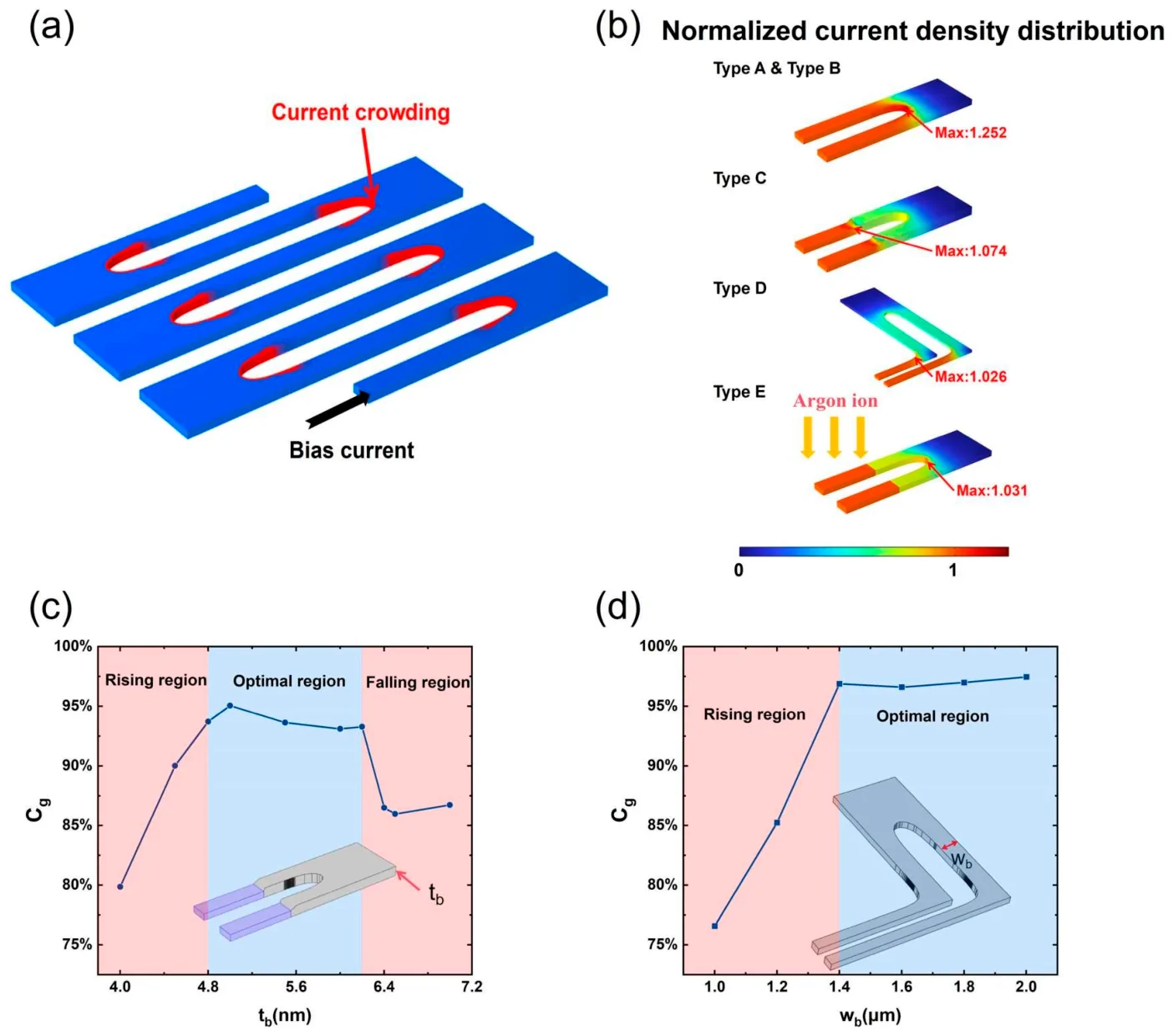
(**a**) Schematic of the current crowding effect. (**b**) The normalized current density distribution of superconducting microstrip with Type A–E. (**c**) The relationship between C_g_ and the bend thickness for superconducting microstrip with Type C. (**d**) The relationship between C_g_ and the bend width for superconducting microstrip with Type D. [78]. Where C_g_ as the ratio of the device’s critical current to that of a straight wire.

The core of the above structural optimization strategies for superconducting microstrips is closely associated with relieving the current crowding effect. Bao et al. [78] systematically evaluated the effectiveness of three mainstream approaches to relieve current crowding via simulation and experimental verification, namely optimizing the curvature of meander corners, regulating local film thickness, and modulating local current density by ion irradiation modification, as shown in Figure 7. Experimental results confirm that all three methods can alleviate current crowding. In particular, ion irradiation applied to the straight regions of superconducting microstrips presents dual superiorities. It suppresses current crowding by adjusting superconducting properties such as increasing sheet resistance and reducing the superconducting energy gap, and meanwhile improves the detection sensitivity of devices.

### 4.2. Vortex Dynamics and Hole Structure Engineering

According to the photon response mechanism of SMSPDs, the vortex dynamics within the superconducting microstrip are correlated with the device photon response. On this basis, Li et al. introduced periodic holes into the superconducting microstrip to optimize the vortex dynamics by modulating the local current density distribution, as shown in Figure 6d [44]. The hole-patterned superconducting microstrips not only reduce the device’s dependence on intrinsic film parameters but also resolve the trade-off between photosensitive area and recovery time. Zhu et al. [79] further investigated the superconducting performance of periodic pinhole structures under magnetic fields, and found that the superconducting microstrips with periodic pinholes exhibit stable critical-current characteristics within specific magnetic-field windows. The dark counts of the device are significantly suppressed while the photon counting performance remains unaffected. Subsequently, Zhu et al. [46] combined TDGL simulations with experimental measurements to reveal two plateau stages of vortex dynamical behavior in periodic rectangular pinhole arrays. The first stage corresponds to the Meissner state, in which a small number of vortices are trapped by the pinholes, but their motion is restricted. Additionally, the critical current (*I*_c_) is insensitive to the applied magnetic field. The second stage is the vortex-flow mixed state, where the pinholes pin vortex–antivortex pairs and guide them into ordered motion along specific directions. These studies indicate that hole structures, by modulating the vortex states in the superconducting microstrip, provide an effective method for suppressing dark counts and improving the current-carrying capability of the microstrips. In addition, a parallel microstrip pair configuration has been developed to expand the photosensitive area while maintaining a low kinetic inductance [71]. This architecture employs two parallel, short microstrips as the basic unit, which can be expanded into multiple parallel pairs through series or parallel connections of basic units, as shown in Figure 6g. This design concept is analogous to that of superconducting nanowire avalanche single-photon detectors (SNAPs). Parallel microstrip pairs based on ultrathin NbRe films have realized single-photon response at a wavelength of 2 μm, demonstrating the great application potential of this structure in mid-infrared detection.

In order to allow the device to reach its intrinsic performance limit, a superconducting “rail” architecture was introduced to both sides of the SNSPD nanowire, as shown in Figure 6i. The rails generate a magnetic field that partially cancels the perpendicular component of the nanowire’s self-field, thereby suppressing the severe current crowding at the wire edges [73]. This mechanism is based on simulations of the London equation, which show that the current density profile can be tuned from edge-peaked to center-peaked as the rail current is varied. Such redistribution increases the energy barrier for vortex entry from the edges, allowing the switching current I_sw_ to approach the depairing current I_dep_. Consequently, the device reaches its intrinsic performance limit, marked by a transition from edge-vortex-dominated dark counts to bulk vortex–antivortex pair unbinding. With the superconducting rail architecture, the device pushes the wire width to 100 μm, lowers the dark count rate by 10 orders of magnitude, and achieves near-unity internal detection efficiency at 4 μm on a 20 μm wide wire. This structure architecture overcomes the Pearl length limitation, paving the way for arbitrarily wide, high-performance superconducting devices.

Table 2 compares the fill factor, DCR performance, fabrication difficulty, and main application scenarios of SMSPDs with different geometrical topologies. The geometrical topologies of SMSPDs significantly affect the fill factor, DCR level, fabrication complexity and applicable directions of the devices. Among them, spiral microstrips and microstrips with candelabra-style bends achieve high fill factors; hole-patterned microstrips feature low dark-count performance with a DCR as low as ~2 Hz. The microbridge structure is easy to fabricate and is mainly used for fundamental mechanism research, and microstrips with pinholes are suitable for vortex dynamics research. Microstrips with candelabra-style bends and microstrips with rails exhibit polarization-insensitive characteristics yet suffer from relatively high fabrication difficulty. In general, meandering microstrips are suitable for developing large photosensitive areas. A high fill factor is usually accompanied by greater fabrication difficulty, while low-DCR structures (e.g., hole-patterned microstrips and HCCB structures) show distinct advantages for specific applications.

## 5. Readout Circuit

Readout circuits are a critical component of SMSPD systems. The circuit for reading photon signals must account for the large biased current and broad thermal dissipation region, which arise from the micrometer-scale linewidth of SMSPD devices. Latching represents an unfavorable electrothermal failure mode inherent to detectors; thus, the latching effect is the most central issue in readout circuit design. The latching phenomenon emerges when the thermal decay timescale of the hot spot exceeds the circuit reset constant [80]. After the formation of the resistive hot zone, the kinetic inductance releases its stored electromagnetic energy in the form of Joule heating, continuously sustaining the resistive region. As bias current gradually flows back into the superconducting strips, thermal relaxation cannot cool the hot spot rapidly enough to restore superconductivity. Consequently, the device remains in a steady resistive state rather than autonomously returning to the superconducting state. Detectors suffering from latching lose their photon detection capability and become insensitive to subsequent optical signals.

### 5.1. Shunt Resistor Method

**Figure 8:**
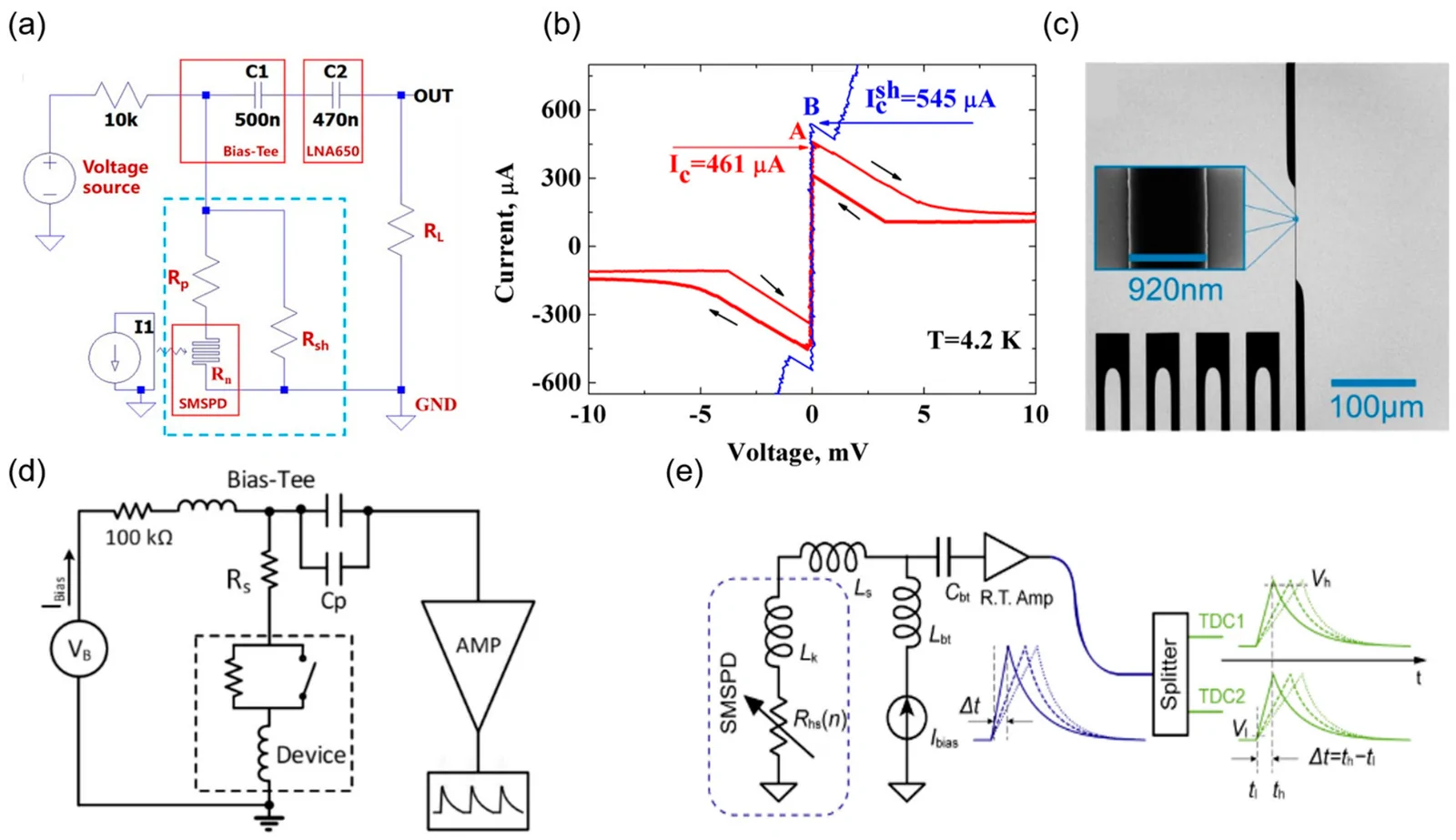
(**a**) Schematic of the readout circuit with a shunt resistor [81]. (**b**) I–V curves of the readout circuit with (blue) and without (red) shunt resistor [33]. (**c**) Schematic of a superconducting microstrip with an on-chip integrated series inductor [34]. (**d**) Schematic of a modified Bias-Tee readout circuit to suppress pulse overshoot of the SMSPDs device [82]. (**e**) Schematic of the dual-channel real-time photon-number readout circuit for the SMSPDs [77].

Owing to the micrometer-scale width of SMSPDs, the localized hot spots generated after photon absorption require heat diffusion across a broader area, which prolongs the heat conduction path and slows down the cooling rate, making the devices more susceptible to latching during the photon response process. As a result, the conventional Bias-Tee readout circuits designed for SNSPDs are no longer compatible with SMSPDs. Shunt resistors integrated with detectors are a strategy to eliminate latching effects in SMSPDs, as shown in Figure 8a. For instance, Korneeva et al. [33] introduced a low-value shunt resistor in parallel with the detector chip. This configuration avoids persistent Joule heating and thermal runaway originating from excessive current accumulation within the quenched region, allowing the device to rapidly quench and recover to the superconducting state. In addition, experimental results reveal that a parallel shunt resistor can enhance the critical current of the detector, as shown in Figure 8b. Vodolazov et al. [16] also adopted the shunt resistance scheme to address latching issues for detectors. In addition, Wang et al. [81] conducted comparative experiments on NbN microstrip detectors to investigate how shunt resistors with different resistance values affect the electrical properties and photoresponse of SMSPDs. The results prove that proper selection of shunt resistance exerts positive effects on broadening the operating bias current range and improving the intrinsic system detection efficiency.

### 5.2. Series Inductor Method

Another approach to suppress the latching effect is to increase the dynamic impedance of the device through a series inductor. When the detector switches to the resistive state after photon absorption, the series inductor limits the instantaneous current variation and redistribution, enabling the detector to recover to the superconducting state. Chiles et al. [56] proposed an on-chip integrated series inductor scheme, in which a meander strip shape with a linewidth larger than that of the superconducting microstrip was fabricated above the detector by using the same superconducting film, as illustrated in Figure 8c. The series inductor effectively retards current changes and suppresses thermal runaway by increasing the total kinetic inductance of the device. Protte et al. [34] also adopted this approach by integrating a series inductor fabricated with the same process onto WSi microstrip detectors in a laser lithography design. This successfully suppressed the latching effect, enabling devices with linewidths ranging from 0.59 µm to 1.43 µm to exhibit saturated internal detection efficiency [57].

### 5.3. Modified Bias-Tee Circuit Method

When the active area of the device becomes sufficiently large, an obvious overshoot phenomenon occurs in the electrical pulse waveform output after single-photon response [83]. This phenomenon also drives the device into a latching state, thereby limiting the upper bounds of the detector’s bias current and photon count rate. The characteristic time of the pulse waveform is τ = *L*_k_/*R*_L_, where *L*_k_ is the kinetic inductance, and *R*_L_ is the load impedance. Consequently, both the kinetic inductance and the load impedance directly affect the voltage pulse characteristics at the device output. To enable the device to operate in an impedance-matched state, Figure 8d presents a modified readout circuit for the SMSPDs to suppress the overshoot phenomenon induced by the increased kinetic inductance owing to the large active area. On the one hand, a shunt capacitor (*C*_p_) is introduced to increase the capacitance of the Bias-Tee. On the other hand, a resistor (*R*_s_) is added in the series branch of the detector to optimize the impedance matching of the transmission line. To verify the influence of different resistance values on the detector pulse shape, Li et al. [82] performed numerical simulations of the electrical characteristics of the readout circuit by using LTspice software 24.0.12 and the Simulation Program with Integrated Circuit Emphasis (SPICE) circuit modeling method.

### 5.4. Photon-Number-Resolving Readout

Significant progress has also been achieved in the development of SMSPD readout circuits for photon-number-resolving (PNR) applications. Kong et al. [77] employed an SMSPD with a large kinetic inductance to naturally stretch the rising edges of output pulses triggered by different photon numbers, thereby extending the temporal separation between different photon-number events beyond the bandwidth limitation of the amplifier. Combined with a dual high-precision time-to-digital converter (TDC), the system enabled high-fidelity real-time photon-number-resolving readout. The detector demonstrated a maximum photon-number resolution capability of up to 10 photons, with identification fidelities reaching 98% and 90% for four- and six-photon events, respectively. Furthermore, this work introduced a dual-channel temporal readout scheme that reduced the data acquisition volume by three orders of magnitude, as shown in Figure 8e. The proposed system was successfully applied to quantum random number generation based on coherent-state parity sampling, demonstrating the significant potential of SMSPDs for quantum information processing applications.

Overall, the design of SMSPD readout circuits requires coordinated optimization across multiple levels. At the device level, appropriate selection of shunt resistors or series inductors is essential to prevent latching effects. At the signal level, improved Bias-Tee circuit designs are required to achieve impedance matching and suppress signal overshoot effects. At the system level, multi-channel multiplexed readout architectures should be developed to support large-area and array-based SMSPD implementations. Similar to SNSPDs, multiplexed readout is one of the key bottlenecks limiting the development of large-scale pixel arrays of SMSPDs. Although multiplexed readout architectures such as row-column [84], time-division [85], frequency-division [86], and thermal-coupling [24] schemes are available for reference to reduce the number of cryogenic cables, such readout architectures are generally limited by readout rate, crosstalk, and defect sensitivity. Meanwhile, the electrical characteristics of SMSPDs pose substantial challenges to multipixel readout implementation. On the one hand, most SMSPDs require external components such as shunt resistors or series inductors to suppress thermal latching. Equipping every pixel with such discrete peripheral elements would increase fabrication complexity, while the thermal load introduced to the cryogenic stage rises sharply and thereby degrades device performance. On the other hand, large-area SMSPD arrays demand high uniformity of critical current and detection efficiency across pixels, for which film-quality homogeneity remains a major challenge to overcome. As SMSPD technology advances toward larger active areas and scalable arrays, innovations in readout circuit design will become one of the key factors determining the further improvement of overall system performance.

## 6. Application Progress

Benefiting from the combined advantages of a large active area, simple fabrication process, low dark count rate, broadband response, and high single-photon detection efficiency, SMSPDs have demonstrated broad application prospects in several emerging fields. The millimeter-scale active area of SMSPDs can reduce the requirements for optical coupling alignment accuracy and enable large numerical aperture (NA) light collection without the need for additional optical front-end components. This feature makes SMSPDs suitable for detecting signal sources with relatively broad spatial distributions.

### 6.1. Quantum Information and Quantum Communication

SMSPDs exhibit two key features for quantum information processing, namely polarization insensitivity and photon-number-resolving capability. While maintaining excellent system detection efficiency, these advantages can significantly improve the key generation rate and transmission distance of quantum key distribution (QKD) systems and play an important role in photonic quantum computing.

In quantum communication applications, the polarization sensitivity of detectors originates from the geometrical anisotropy of the meander structures. Therefore, polarization controllers are typically required to maintain optimal detection efficiency, which not only increases system losses but also introduces potential security vulnerabilities in phase-encoding QKD protocols. To address this issue, Reddy et al. [72] developed an SMSPD based on a candelabra-shaped meander structure combined with a WSi superconducting thin film. The fabricated detector exhibited a strip width of 0.51 μm and a high fill factor of 0.91. At a wavelength of 1550 nm, the detector achieved an extremely low polarization sensitivity of 1.02 ± 0.008, while maintaining a system detection efficiency above 91.8% over a 40 nm bandwidth. This approach overcomes the efficiency loss and potential security vulnerabilities caused by polarization control in conventional schemes, providing a promising detector platform for highly robust and efficient practical quantum communication systems.

**Figure 9:**
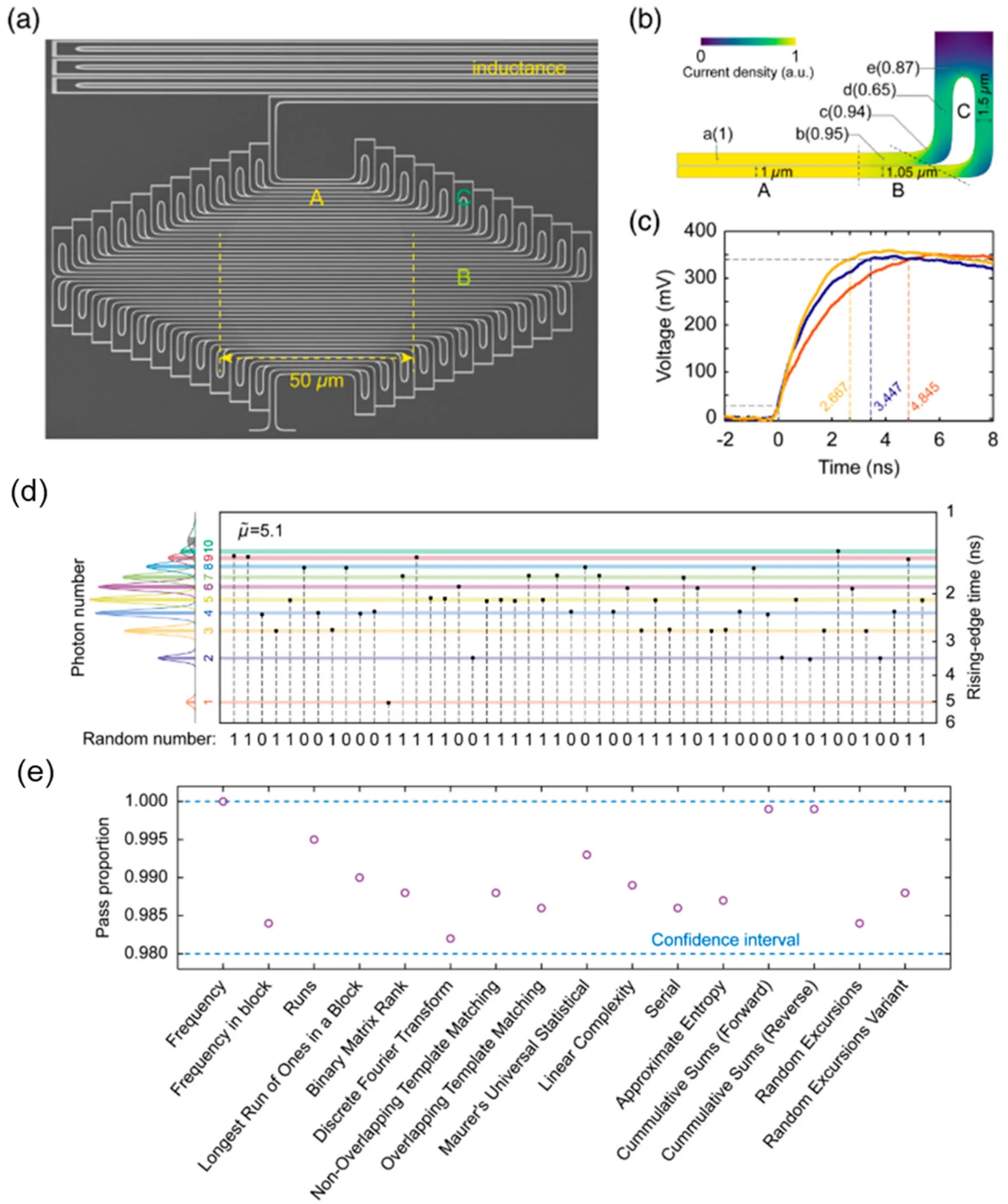
(**a**) Scanning electron micrograph of the SMSPD. (**b**) The simulated current density distribution of superconducting microstrips. (**c**) Rising edge of representative output waveforms for one, two and three photon detection events. (**d**) Operating principle of the quantum random number generation. (**e**) Results of the generated random bit sequences [77].

In quantum information processing, photon-number-resolving capability is a fundamental requirement for linear optical quantum computing, boson sampling, and quantum metrology. Endo et al. [87] characterized superconducting nanostrip photon-number-resolving detectors using quantum detector tomography (QDT), demonstrating a maximum photon-number resolution of five photons without employing detector arrays. Kong et al. [77] further exploited the large kinetic inductance effect of SMSPDs to achieve high-fidelity resolution of up to 10 photons on a single detector by analyzing the differences in the rising-edge temporal characteristics of pulses triggered by different photon numbers. Based on this capability, they further demonstrated a quantum random number generator based on coherent-state photon-number parity sampling. Figure 9 illustrates the overall device architecture and the generation and testing of quantum random numbers. The device adopts a candelabra-style structure, in which the entire microstrip is divided into a detection section A, a connecting section B, and a bending section C, as shown in Figure 9a. Figure 9b demonstrates the simulation of current density in different regions of the microstrip. The detection section A exhibits the highest current density, whereas the connecting and bent sections show lower current-density values. This indicates that the candelabra-style segmented design can effectively confine high current density within the detection area, while preventing premature quenching caused by current crowding at the bent regions. Figure 9c shows that the pulse rise times corresponding to one-, two-, and three-photon events are 4.845, 3.447, and 2.667 ns, respectively. It can be seen that the wide-microstrip structural design endows SMSPDs with large kinetic-inductance characteristics. The rising edges of output pulses for different photon events are significantly stretched, so that the photon number can be encoded into the rise times. Figure 9d illustrates the complete mapping among photon number, rise-edge time, parity and random bit. Figure 9e further presents the NIST randomness test results for 1000 sets of random bit streams, with each set containing 10^6^ bits. The pass ratio of all tests falls within the confidence interval of 0.98–1, demonstrating that the output bits possess good randomness. Therefore, SMSPDs have the capability of high-fidelity photon-number resolution, which can be utilized for robust quantum random-number generation and provides a concise yet effective detector platform for quantum information processing.

### 6.2. Dark Matter Detection

**Figure 10:**
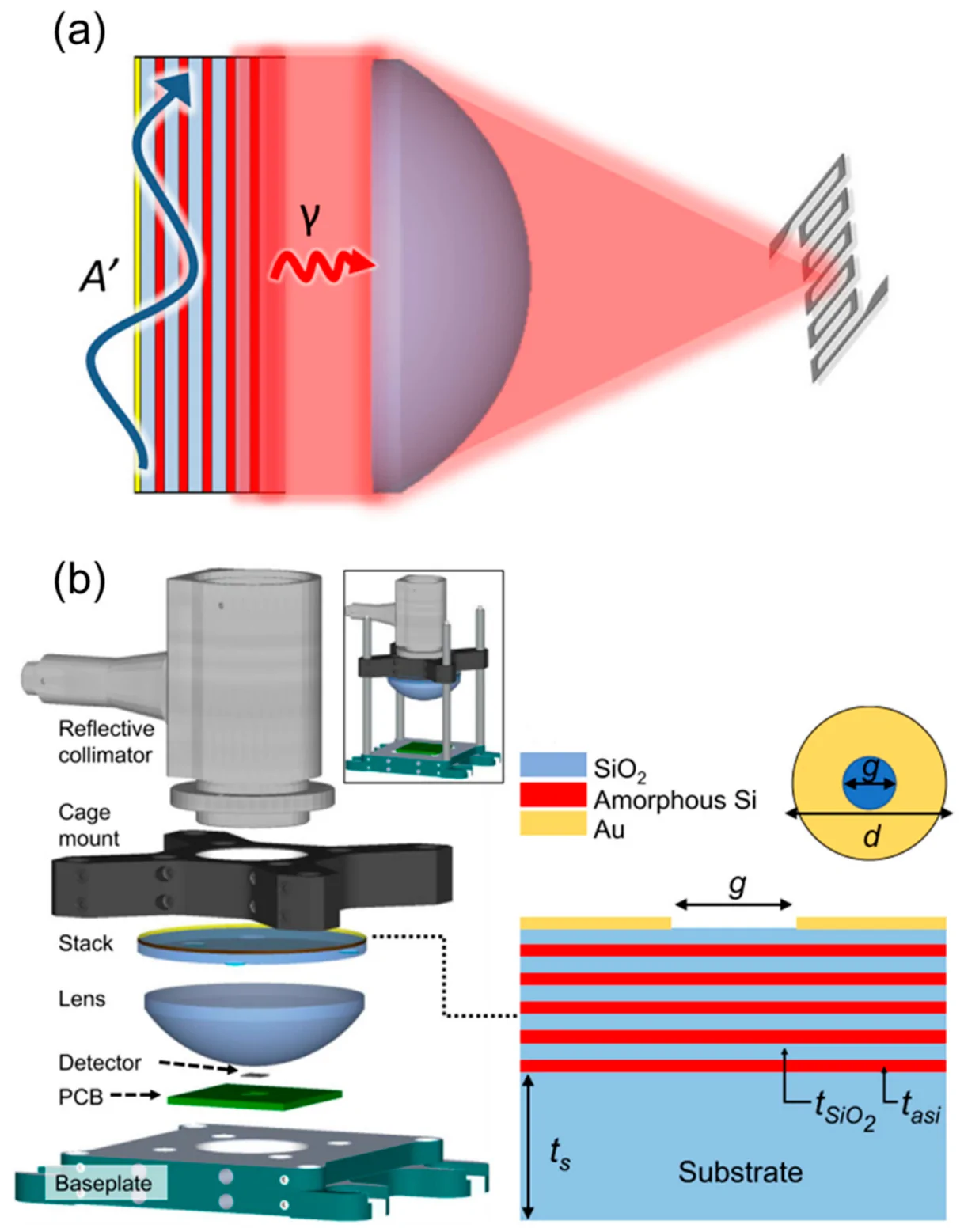
(**a**) Schematic of the LAMPOST experiment. The dark photon dark matter field, denoted A′, converts into photons within a layered dielectric target. Photons are focused by a lens onto a SNSPD detector. (**b**) Schematic of the components of the LAMPOST prototype haloscope apparatus and detailed views of the dielectric stack target [89].

Dark matter detection represents one of the most important frontier research topics at the intersection of contemporary particle physics and astrophysics. Hochberg et al. [88] pioneered a scheme that utilizes WSi superconducting nanowires simultaneously as both target materials and sensors for the direct detection of sub-GeV dark matter. Using WSi superconducting nanowires as prototype detectors, they experimentally demonstrated the sensitivity of detectors to electron recoils and their ultra-low dark count rates, establishing the most stringent terrestrial constraints on sub-eV dark photon absorption to date. Furthermore, Chiles et al. [89] employed SNSPDs in the optical cavity-based dark matter search experiment, which is named the Light A′ Multilayer Periodic Optical SNSPD Target (LAMPOST) experiment, improving the constraints on the kinetic mixing parameter of dark photons within the mass range of 0.7–0.8 eV by a factor of two. Figure 10 shows the concept of the LAMPOST, including schematics of the LAMPOST experimental framework and its key components. They further pointed out that future large-area superconducting detectors, such as SMSPDs, could substantially expand the accessible parameter space for dark photon and axion dark matter searches.

SMSPDs exhibit particularly prominent potential in dark matter detection due to their scalable active area and low dark count rates. In contrast to SNSPDs, which are limited to active areas on the scale of hundreds of micrometers, SMSPDs can be fabricated into large-area detector arrays ranging from millimeter to square-centimeter scales through direct laser writing or ultraviolet photolithography. This scalability enables a substantial increase in target mass, thereby addressing the urgent requirement for large-area sensors in low-background rare-event detection experiments.

Luskin et al. [53] fabricated the first eight-pixel SMSPD array with a total active area of 1 mm^2^. The detector achieved saturated quantum efficiency at the 1064 nm wavelength and a dark count rate on the order of 10^−1^ Hz, providing a scalable solution for low-mass dark matter searches. Wang et al. [65] further conducted systematic temperature-dependent characterization of a four-pixel SMSPD array. The detector was characterized over a temperature range from 0.2 K to 1.2 K, with measurements of quantum efficiency, timing jitter, and dark count rate. The results showed that the detector maintained saturated quantum efficiency over a broad spectral range from 635 nm to 1650 nm, with a timing jitter of approximately 160 ps and dark count rates as low as 10^−2^ Hz. This work also presented the first systematic investigation of dark count coincidence events between pixels in SMSPD arrays. An excess of correlated counts beyond the expected accidental coincidence rate was observed in the low-bias-current regime. The insensitivity of these correlation signals to the coincidence time window suggests that they may originate from a common physical source, such as cosmic-ray muons. This finding provides critical insights for the identification and rejection of background events in large-scale array-based experiments.

### 6.3. High-Energy Particle Detection

**Figure 11:**
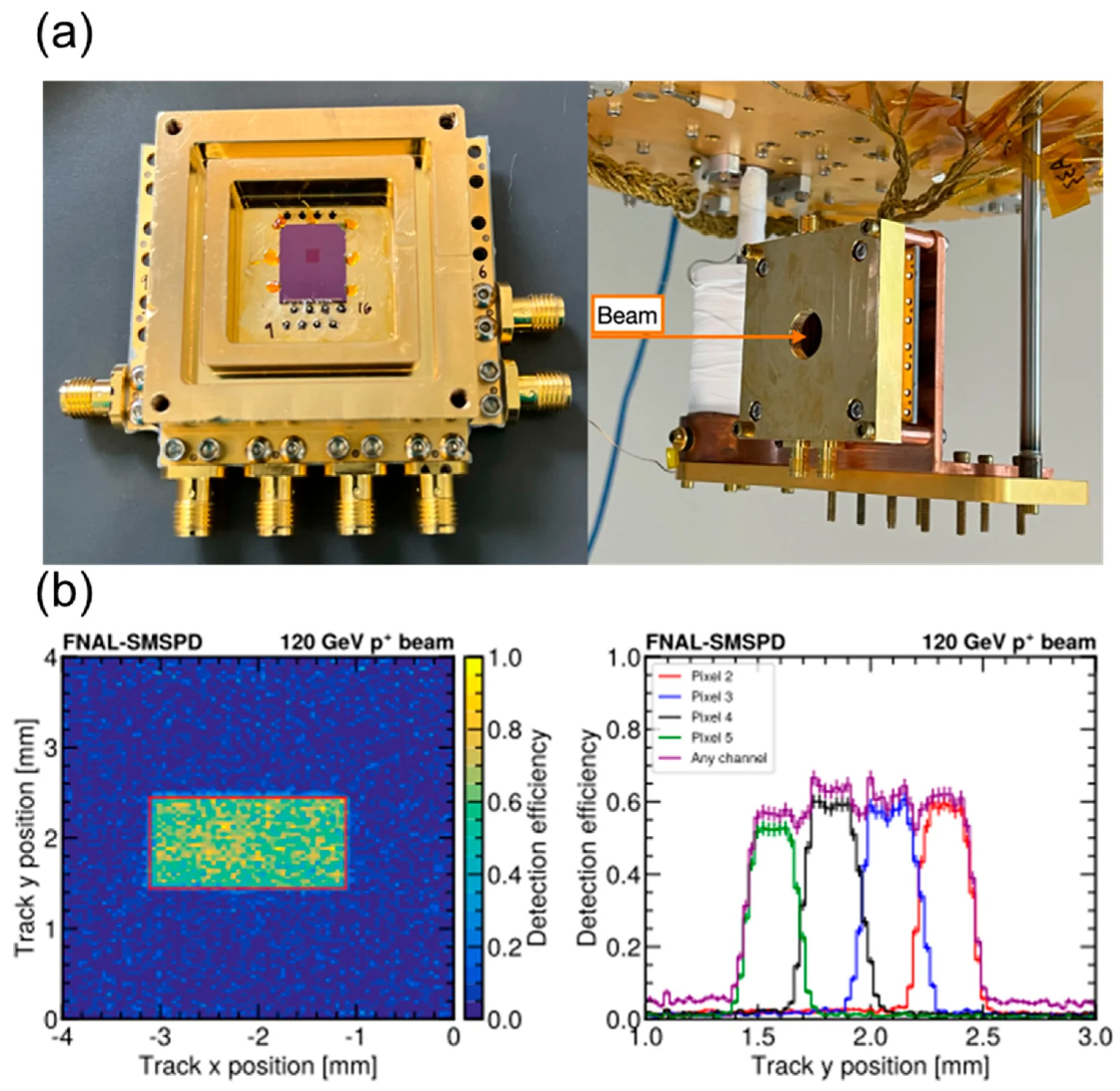
(**a**) Photograph of the packaged SMSPD device. (**b**) Detection efficiency of the 4-pixel SMSPD, which the detection efficiency as a function of the incident proton track position [54].

High-energy particle detection refers to the detection, identification, and characterization of high-energy charged or neutral particles with energies typically in the giga-electron-volt (GeV) range and above. The fundamental principle relies on the interaction between energetic particles and the detector medium, where the energy deposited by incident particles is converted into measurable electrical signals. After amplification and digitization, these signals are used to reconstruct key particle parameters, including energy, momentum, position, and arrival time. In 2025, Peña et al. from Fermi National Accelerator Laboratory demonstrated the capability of large-area WSi-SMSPD arrays with a total active area of 2 × 2 mm^2^ for high-energy particle detection. The package diagram of the SMSPD device is shown in Figure 11a. The SMSPD channels achieved detection efficiencies above 40% at bias currents ranging from 10 to 13 μA, while maintaining dark count rates below 1 Hz and a timing resolution of 1.15 ns [54]. Figure 11b shows the detection efficiency distribution of the device with respect to the incident proton track position. The experiment successfully detected 120 GeV protons, eight GeV electrons, and pion beams. This work represents the first direct detection of high-energy particles using SMSPDs, providing a novel high-sensitivity detection approach for particle physics experiments, such as time-of-flight measurements and forward scattering studies.

**Figure 12:**
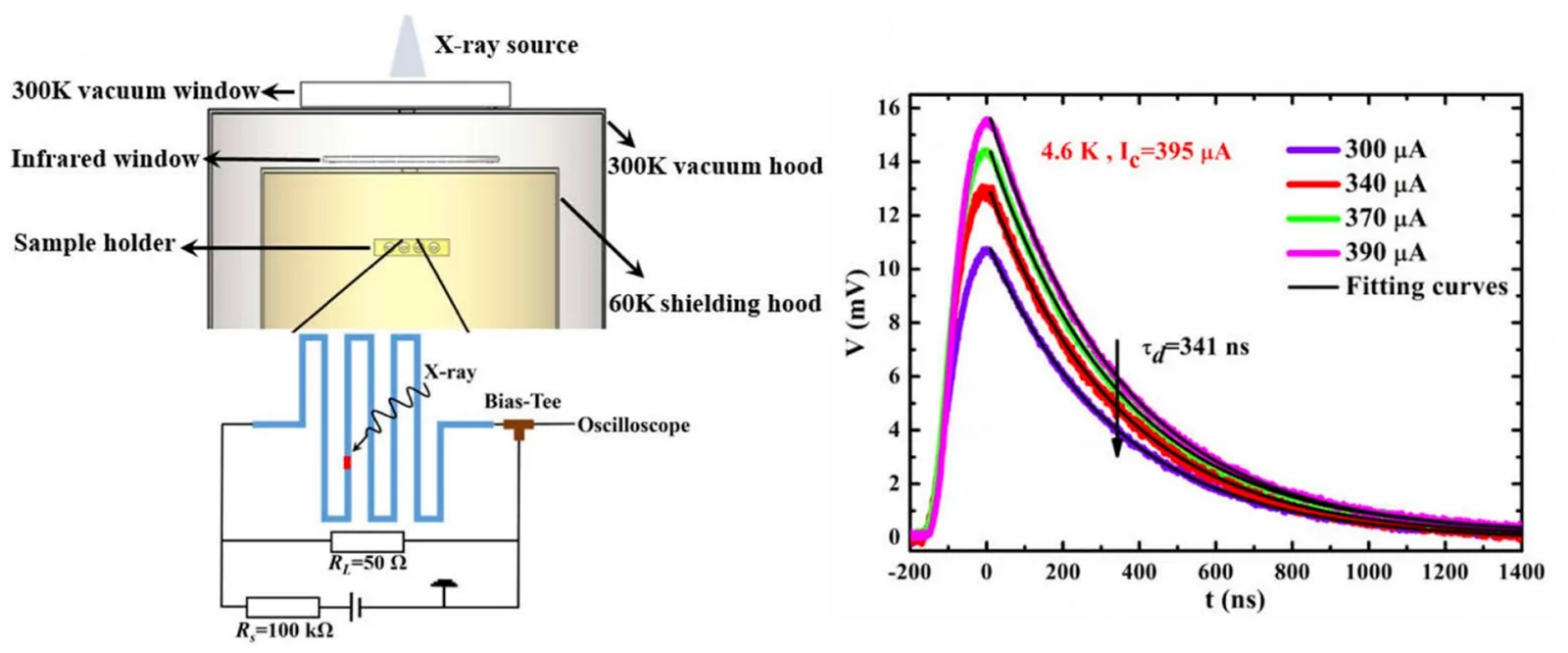
Schematics of the experimental setup and the readout circuit diagram (**left**). Signal pulses of a Fe-55 isotope source at various bias currents at 4.6 K (**right**) [90].

In addition, SMSPDs have also shown promising potential in X-ray detection applications. Yang et al. [90] investigated the response of large-area TaN-SMSPDs to 5.9 keV and 10 keV X-rays, as shown in Figure 12. The results demonstrated that a higher bias current leads to improved energy resolution, while the dark count rate remained as low as approximately 10^−4^ Hz at 90% of the critical current. Experimental results confirmed that TaN-SMSPDs can detect X-rays over a wide range of bias currents and operating temperatures, and can distinguish X-ray photons with different energies under optimized operating conditions. These results establish a foundation for the application of SMSPDs in X-ray detection fields, including material analysis, security inspection, medical imaging, and astrophysics.

### 6.4. Single-Photon Imaging

Single-photon imaging relies on detectors for sensitive detection and time tagging of photons. Through techniques such as time-correlated single-photon counting (TCSPC), it enables efficient reconstruction of target scenes under ultra-low light conditions. The large active area of SMSPDs makes them naturally suited for imaging systems based on multimode fiber coupling or direct spatial sampling, significantly improving light collection efficiency and reducing the engineering complexity of optical alignment.

**Figure 13:**
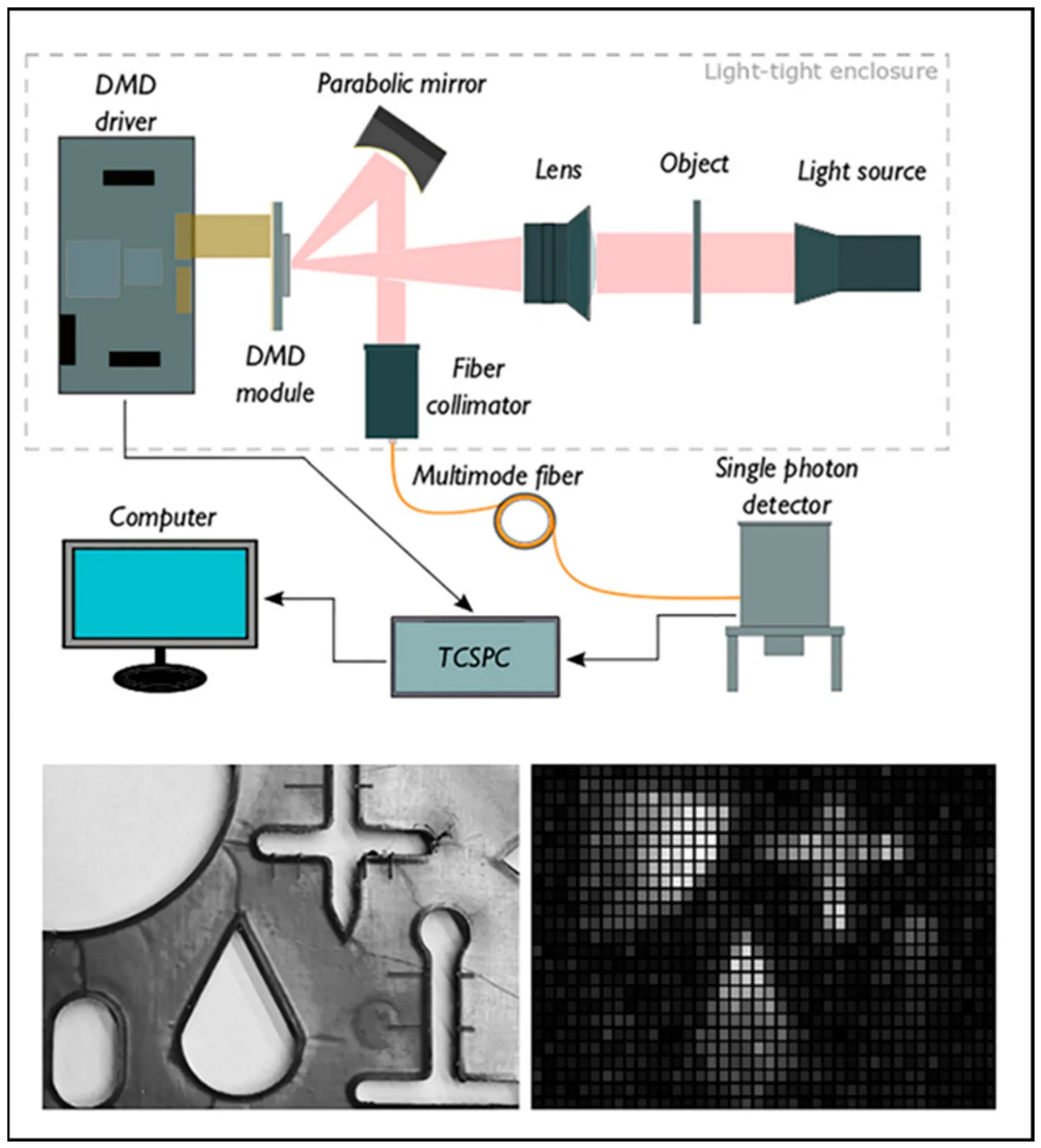
Schematics of the single-pixel camera experimental setup (**top**). Test object and image from a single-pixel camera (**bottom**) [91].

Shcherbatenko et al. [91] developed an NbN-SMSPD with an effective area of 50 × 50 μm and coupled it to a multimode fiber with a 50-µm core diameter. Figure 13 illustrates the single-pixel camera experimental setup and the image result. The system employed a digital micromirror device to generate temporally modulated spatial light patterns, which encoded the two-dimensional target structure into a one-dimensional temporal intensity signal in a single-pixel computational imaging scheme. The signal was acquired by a single detector without spatial resolution, and the image was reconstructed by solving a system of linear equations. The target image was successfully reconstructed under ultra-weak light conditions, with an average of approximately three photons per pixel at a resolution of 25 × 40 effective pixels.

**Figure 14:**
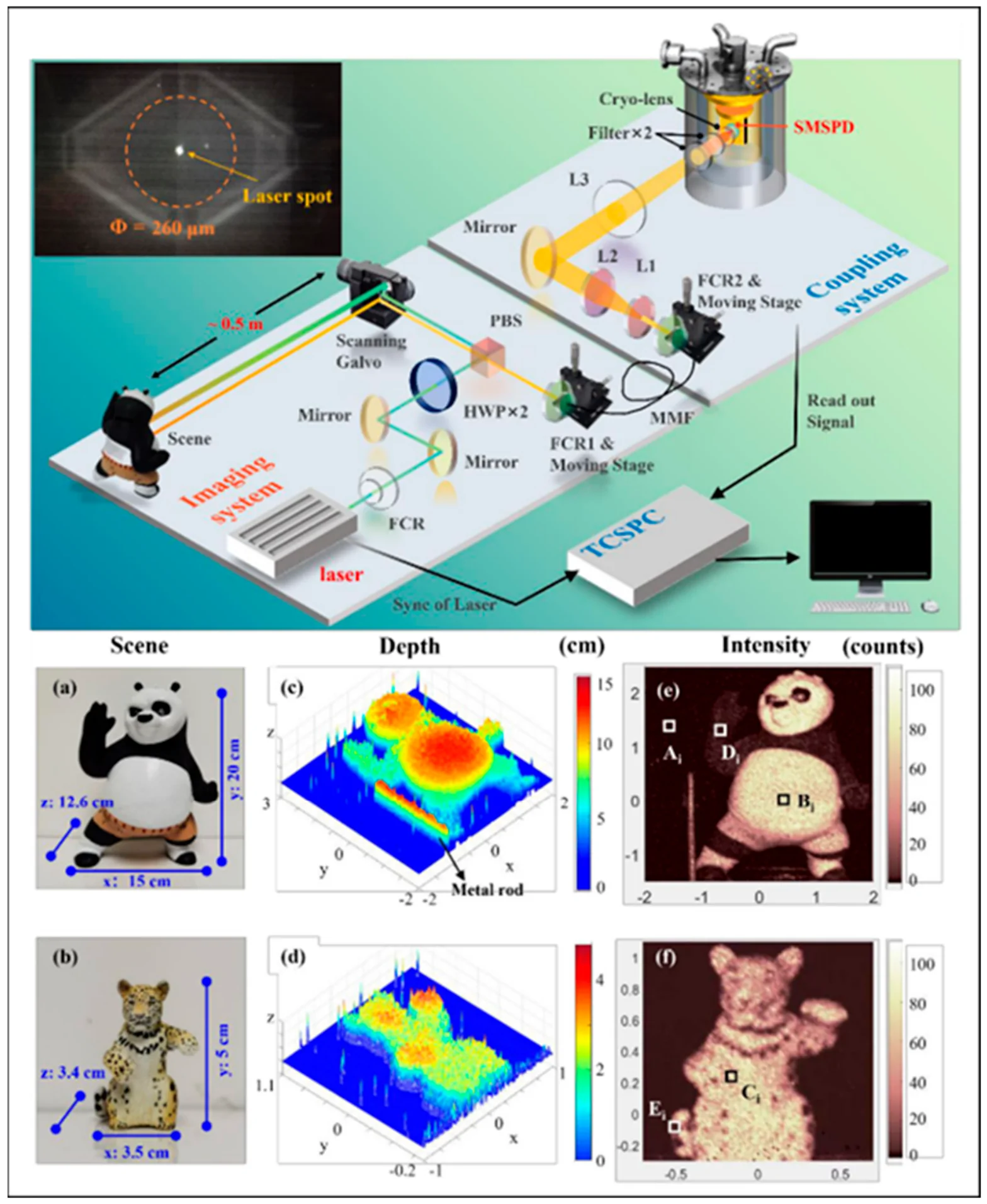
Schematic of the time-of-flight imaging experiment setup, including a coupling system and an imaging system (**top**). Photos of the scenes (**bottom**): (**a**,**b**) Imaging scenes. (**c**,**d**) the corresponding reconstructed depth. (**e**,**f**) intensity images [76].

Zhang et al. [76] investigated photon-counting time-of-flight (ToF) imaging by exploiting the excellent timing resolution of large-area SMSPDs. By measuring the time interval between the emission of a laser pulse and its return to the detector after reflection from target objects, the system reconstructed both depth and intensity information of the target scene. The SMSPD featured an active area with a diameter of 260 μm, and under free-space coupling at an operating temperature of 2.0 K, the detector achieved a system detection efficiency of about 2% at 1550 nm and a dark count rate of 5 kHz. Figure 14 presents experimental results showing that, for a panda toy with dimensions of approximately 20 × 15 × 5 cm^3^, the depth image clearly resolved a depth difference of approximately 6 cm between the hand and abdomen regions, achieving a depth resolution of approximately 2.5 cm, consistent with the measured timing jitter of 171 ps. The reconstructed intensity image exhibited a maximum signal-to-background ratio (SBR) exceeding 80.

These results indicate that SMSPDs overcome the active-area limitations of conventional devices, enabling direct free-space or multimode-fiber coupling, substantially reducing optical alignment requirements, and improving photon collection efficiency. Furthermore, the outstanding timing resolution of SMSPDs enables intrinsic three-dimensional depth sensing, providing unique advantages for LiDAR and 3D scene reconstruction.

## 7. Conclusions and Perspectives

As an important emerging member of the superconducting single-photon detection family, SMSPDs have achieved systematic progress in several key areas since their theoretical proposal and experimental demonstration, including the understanding of operating mechanisms, expansion of material systems, optimization of device architectures, development of readout circuits, and exploration of advanced applications. This review has systematically summarized these five core aspects and provides an outlook on future development directions.

(1) Breakthroughs in new materials

Currently, SMSPDs mainly rely on conventional superconducting thin films such as NbN, MoSi, and WSi. With advances in high-temperature superconducting materials including MgB_2_ and YBCO, the operating temperature of SMSPDs is expected to extend from below 4 K to 20 K or even above 77 K, which can substantially reduce the requirements and costs of cryogenic systems. Future research should focus, on the one hand, on precise control of the properties of low-energy-gap superconducting materials, and on the other hand, on overcoming the challenges of uniform fabrication of high-temperature superconducting films, enabling their detection efficiency and operating temperature to reach levels comparable to those of SNSPD devices.

(2) Enhancement of optical absorption efficiency

Typically, the optical absorption efficiency of SMSPDs is limited by the low fill factor, with some structures exhibiting fill factors below 0.5. Adopting superconducting microstrips with topological configurations can improve the fill factor of SMSPDs. Alternatively, integrating optical cavity structures or adopting plasmonic enhancement techniques is expected to improve the photon absorption efficiency without sacrificing the detector’s active area or response speed.

(3) Large-scale array fabrication and readout

The active area of individual SMSPD pixels has already reached the millimeter scale. However, applications in high-energy physics and dark matter detection require the development of large-scale detector arrays with areas reaching square centimeters or even larger. Such large-area arrays face significant challenges, including material uniformity, scalable readout multiplexing, and crosstalk suppression. These challenges will require the integration of advanced microwave readout technologies, such as frequency-division multiplexing and time-division multiplexing, to enable scalable detector architectures.

(4) Expansion of the detection wavelength range

The detection wavelength range of SMSPDs has currently been extended from soft X-rays to the mid-infrared region (approximately 2 μm). However, their performance in the mid- to far-infrared range (3–12 μm) remains limited. By employing superconducting materials with lower energy gaps and optimizing vortex dynamics parameters, SMSPDs may further extend their detection capability toward the mid-infrared regime, opening new opportunities in astronomical spectroscopy and chemical molecular sensing.

In summary, SMSPDs combine the high-performance characteristics of SNSPDs with the fabrication simplicity and functional versatility enabled by wider superconducting microstrips. These advantages provide SMSPDs with highly competitive application potential in frontier fields including quantum information, fundamental physics experiments, and single-photon imaging. With continuous advances in materials science, nanofabrication technologies, and cryogenic electronics, SMSPDs are expected to transition from laboratory prototypes toward practical engineering applications in the near future and become one of the key platforms in the next generation of superconducting single-photon detection technologies.

## Figures and Tables

**Table 1:** Key parameters of the superconducting materials used in SMSPD fabrication.

Material	Thickness	Superconducting Critical Temperature	Sheet Resistance	Diffusion Coefficient	Published Year	Reference
NbN	5.7 nm	9 K	550 Ω/□	0.4	2021	[49]
NbN	7 nm	6.4 K	1036 Ω/□	0.5	2021	[50]
NbN	5 nm	8.3 K	1250 Ω/□	0.5	2023	[51]
W_52_Si_48_	2.1 nm	2.9 K	882 Ω/□	-	2020	[52]
W_42_Si_58_	4.7 nm	1.8 K	750 Ω/□	-	2023	[53]
W_50_Si_50_	3 nm	2.8 K	1200 Ω/□	-	2025	[54]
MoSi	3 nm	4.1 K	585 Ω/□	0.47	2020	[55]
Mo_75_Si_25_	2.1 nm	3.3 K	1015 Ω/□	0.48	2021	[56]
Mo_80_Si_20_	5 nm	4.2 K	268 Ω/□	-	2022	[44]
NbTiN	5.2 nm	7 K	1069 Ω/□	0.52	2025	[57]
Nb_15_Re_85_	4 nm	5.15 K	250 Ω/□	-	2022	[58]
NbReN	7.5 nm	4.4 K	400 Ω/□	0.7	2024	[59]
MgB_2_	12.5 nm	37.6 K	16.2 Ω/□	0.98	2023	[60]
YBCO	100 nm	89.6 K	110 Ω/□	-	2020	[61]

**Table 2:** Comparison of fill factor, DCR performance, fabrication difficulty and main application scenarios for SMSPDs with different geometrical topologies.

Topologies	Fill Factor	DCR	Fabrication Difficulty	Applicable Scenarios
Microbridge [33]	-	-	Easy	Fundamental mechanism research
Meandering microstrip [55]	0.23	~10^3^ Hz	Moderate	Large-area single-photon detection
Spiral microstrip [50]	0.8	∼200 Hz	Moderate–high	Quantum optics and LIDAR
Hole-patterned microstrip [44]	0.5	∼2 Hz	Moderate–high	low DCR and short reset time detection
Microstrip with candelabra- style bend [72]	0.91	~10^4^ Hz	High	Polarization-insensitive and high- efficiency
Microstrip with HCCB [64]	-	∼4 Hz	Moderate	Polarization-insensitive, large-scale photonic quantum systems
Parallel microstrip pairs [71]	-	-	Moderate	Mid-infrared detection and LIDAR
Microstrip with pinholes [46]	-	-	Moderate–high	Experimental for vortex dynamics
Microstrip with rails [73]	-	-	High	Polarization-insensitive detection

## Data Availability

No new data were created or analyzed in this study. Data sharing is not applicable to this article.
